# Colonial, more widely distributed and less abundant bird species undergo wider population fluctuations independent of their population trend

**DOI:** 10.1371/journal.pone.0173220

**Published:** 2017-03-02

**Authors:** José J. Cuervo, Anders P. Møller

**Affiliations:** 1 Department of Evolutionary Ecology, Museo Nacional de Ciencias Naturales, CSIC, Madrid, Spain; 2 Ecologie Systématique Evolution, Université Paris-Sud, CNRS, AgroParisTech, Université Paris-Saclay, Orsay, France; Friedrich-Schiller-Universitat Jena, GERMANY

## Abstract

Understanding temporal variability in population size is important for conservation biology because wide population fluctuations increase the risk of extinction. Previous studies suggested that certain ecological, demographic, life-history and genetic characteristics of species might be related to the degree of their population fluctuations. We checked whether that was the case in a large sample of 231 European breeding bird species while taking a number of potentially confounding factors such as population trends or similarities among species due to common descent into account. When species-specific characteristics were analysed one by one, the magnitude of population fluctuations was positively related to coloniality, habitat, total breeding range, heterogeneity of breeding distribution and natal dispersal, and negatively related to urbanisation, abundance, relative number of subspecies, parasitism and proportion of polymorphic loci. However, when abundance (population size) was included in the analyses of the other parameters, only coloniality, habitat, total breeding range and abundance remained significantly related to population fluctuations. The analysis including all these predictors simultaneously showed that population size fluctuated more in colonial, less abundant species with larger breeding ranges. Other parameters seemed to be related to population fluctuations only because of their association with abundance or coloniality. The unexpected positive relationship between population fluctuations and total breeding range did not seem to be mediated by abundance. The link between population fluctuations and coloniality suggests a previously unrecognized cost of coloniality. The negative relationship between population size and population fluctuations might be explained by at least three types of non-mutually exclusive stochastic processes: demographic, environmental and genetic stochasticity. Measurement error in population indices, which was unknown, may have contributed to the negative relationship between population size and fluctuations, but apparently only to a minor extent. The association between population size and fluctuations suggests that populations might be stabilized by increasing population size.

## Introduction

Temporal variability in population size, and the causes and consequences of such variability, are central topics in ecology and population biology [1–4], and, therefore, also in conservation biology [5–7]. For example, greater probability of extinction can be expected for populations having higher temporal variability in size [8–10]. Temporal variability in population size has been studied at both intraspecific and interspecific levels (e.g. [11]), and factors affecting this variability, generally classified into environmental effects or endogenous density-dependent processes, are numerous (e.g. [12–19]) and interact in complex ways [20–21].

Temporal variability in population size is often estimated as inter-annual variability, which can be quantified by standard statistics such as the coefficient of variation or the standard deviation [22]. However, inter-annual variability in population size can be split into two components, in the same way as done for size variation in morphological traits [23,24]. If estimates of population size are regressed on years, high levels of inter-annual variability can either arise from large (positive or negative) slopes, from large dispersion of observations around the regression line, i.e., a large standard error of the estimate (SEE, the square root of the residual mean square from the regression [25]), or both. Slopes provide an estimate of population trends, with positive slopes for populations that are increasing in size and negative slopes for populations that are decreasing. In contrast, SEE provides an estimate of population fluctuations around the trend, i.e., temporal variability in population size independent of population trend. These two components of variation provide qualitatively different information and should therefore be studied independently of each other. Despite the large amount of research focusing on variability in total population size (see references above), or on population trends (e.g. [26–28]), temporal variability in population size independent of population trend has rarely been specifically studied.

In a previous study on variability in breeding population size in European bird species [29], we found that populations fluctuated more widely (independent of latitude and population trend) at the edges of the breeding distribution range. Interestingly, temporal variability in population size also differed markedly among species [29]. If populations of some species fluctuate more widely than others, we can speculate that certain ecological, demographic, life-history or genetic characteristics of these species will be related to the degree of fluctuation. Specifically, we hypothesise that the magnitude of population fluctuations might be related to (i) abundance: more abundant species fluctuating less [30], possibly because the effects of demographic stochasticity increase at small population sizes [31]; (ii) distribution range: species with larger geographical ranges fluctuating less due to the positive relationship between population size and distribution range [32,33]; (iii) spatial heterogeneity in distribution: more homogeneously distributed species fluctuating less because more abundant species are found at a higher proportion of available sites [34], and because susceptibility to environmental changes increases the heterogeneity in distribution [35]; (iv) migration: migratory species fluctuating less than resident ones [36], if migration ensures milder or more predictable winter conditions [37]; (v) dispersal: species with longer dispersal distance fluctuating less because dispersal tends to override local factors and synchronize temporal variability among populations [38], but might also fluctuate more if mortality risk increases with dispersal distance [39], i.e., dispersal might stabilize or destabilize population dynamics depending on specific features of the population [40]; (vi) adult survival: species with higher survival rates fluctuating less [30], possibly because demographic stochasticity decreases with survival [41]; (vii) predation: species under weaker predation pressure fluctuating less due to the usual strong effect of predation on adult mortality (e.g. [42]); (viii) parasitism: less parasitized species fluctuating less if parasites destabilize population dynamics [43], but might also fluctuate more if multiple parasitism occurs and parasites compete with each other [44]; (ix) reproductive rate: species producing less offspring fluctuating less [30], maybe because reproductive rate is positively associated with environmental stochasticity [45], although the opposite might also be expected according to the positive relationship between reproductive rate and population size [46]; (x) body size: larger species fluctuating less if large species show low reproductive rate and high adult survival [47], but might also fluctuate more if abundance declines with body mass [48]; (xi) relative brain size: species with larger brains fluctuating less because the cognitive abilities associated with large brains increase survival [49] and population size [50]; (xii) sexual dichromatism: monochromatic species fluctuating less because sexual selection, the strongest predictor of sexual dimorphism [51,52], increases variation in reproductive success, with a negative effect on effective population size [53], and decreases survival [54]; (xiii) relative number of subspecies: species with less subspecies per unit area fluctuating less due to the negative relationship between number of subspecies and dispersal [55]; (xiv) coloniality: non-colonial species fluctuating less because coloniality seems to be associated with patchy and unpredictable environments, i.e., with environmental stochasticity [56]; (xv) habitat: terrestrial species fluctuating less because aquatic habitats are often patchily distributed (e.g. freshwater habitat) or associated with unpredictable resources (e.g. marine habitat [57]); and (xvi) genetic variability: species with larger levels of genetic variability fluctuating less because the bottleneck and genetic drift associated with the low phases of wide fluctuations [58,59] would reduce genetic variability, and also because any reduction in genetic variability would decrease population mean fitness and size [60,61].

From a conservation biology perspective, an important feature of species is their ability to respond to current global change. One example of such ability is the earlier arrival of migratory species to the breeding grounds in response to global warming [62]. The inability of some species to advance their spring migration seems to have a negative effect on population trends [27]. Another example is the colonisation of urban areas in response to the conversion of natural habitats and farmland to urban environments, an ability that is strongly associated with population size and density [63]. As we did above with other species characteristics, we can also hypothesise that the ability of species to respond to global change will be related to the degree of population fluctuations. In general, smaller fluctuations would be expected when species respond adequately to environmental changes, because an appropriate response will have a positive effect on abundance. Specifically, we hypothesise that the magnitude of population fluctuations might be related to (xvii) advancement of spring migration phenology: species advancing their migration more fluctuating less; and (xviii) colonisation of urban areas: species expanding their range into urban areas fluctuating less than species that have not become urbanised.

The aim of this study was to test the hypothesis that interspecific differences in the pattern of population fluctuations (temporal variability in population size independent of population trend) are related to species-specific ecological, demographic, life-history or genetic characteristics (see predictions above). European breeding bird species were used as study subjects because birds are one of the best studied classes of animals, particularly in Europe, with extensive information on their biology and ecology readily available in handbooks (e.g. [64]). Moreover, bird census programmes in a number of European countries have compiled annual time series of population sizes for many bird species, and this information is often freely available. National bird census programmes have been coordinated by the Pan-European Common Bird Monitoring Scheme (European Bird Census Council; http://www.ebcc.info/pecbm.html), providing the opportunity to standardize methodologies and thus making censuses from different countries comparable.

## Methods

### Estimates of population fluctuations in bird species

Population size estimates of European bird species were obtained from websites and persons responsible for the Pan-European Common Bird Monitoring Scheme in 12 European countries: Austria, Czech Republic, Denmark, Finland, France, Germany, Hungary, Netherlands, Norway, Poland, Spain, and Sweden [29]. Although we attempted to obtain information from all European countries, it was impossible because in some countries this scheme had started very recently or had not started yet, or because information was unavailable upon request. Population size estimates from some countries or regions could not be used in this study owing to incomplete information (e.g. Wallonia), or because bird censuses were done only in one type of habitat (e.g. Latvia). All species with available information were included except Canada goose *Branta canadensis* and common pheasant *Phasianus colchicus* because they are introduced in Europe [64].

The estimates of population size that we obtained were population indices, which had been calculated in the same way in all European countries following recommendations made by the European Bird Census Council (EBCC; see http://www.ebcc.info). Specifically, population indices had been calculated using TRIM (Trends and Indices for Monitoring Data), a software package developed for the analysis of time series of counts with missing observations. Information about the programme TRIM can be found at [65], and for an example of the use of population indices, see [66]. Population indices are always relative values, because they are calculated by standardizing individual counts (or count estimates) to a value of one in a particular year while the rest of the years indicate a value relative to the reference year. For example, if the population index in a year is two for a particular species, it means that the population size of that species is twice the value in the reference year. Number of bird species per country ranged from 58 to 170, mean number of years with data in every country from 7 to 33.3, and last year with information on population size from 2004 to 2008 [29]. Population indices were obtained for a total of 231 bird species and 1189 populations (i.e., country x bird species combinations). Number of countries with information for each species differed greatly, ranging from only one country in 55 species to all 12 countries in 22 species [29]. The search for information on population indices was finished by 8^th^ June 2009. Once population indices were compiled, they were regressed on years for every population, and the SEE (calculated as the square root of the residual mean square from the regression) was considered our estimate of population fluctuations around the trend. SEE represents the average distance that the observed values deviate from the regression line, and gives an indication of the accuracy of predictions made by the regression function (see [25], p. 271). We were particularly interested in estimates of population fluctuations that did not include population size variation due to population trend, and we consider that SEE was the best parameter fulfilling that requirement (see Introduction). For a detailed description of the methods used to obtain population indices and to estimate the degree of fluctuation for each population, see [29].

The next step was to calculate an estimate of population fluctuations for each bird species, but, in addition, we were also interested in having these estimates controlled for a number of parameters potentially affecting temporal variability in population size. To this end, we performed a General Linear Model (GLM) with SEE of the regression of population indices on years for every population as the dependent variable (this variable was (log_10_(x) + 1.7) ^0.4^-transformed to achieve an approximately normal distribution), species as a fixed factor, and the parameters presumably affecting temporal variability in population size as independent variables. These parameters were: (i) population marginality within the breeding distribution range, ranging from 0 (central population) to 1 (marginal population) (central populations fluctuate less [29]); (ii) population latitude (in degrees) (southern populations increase less in size [29]); (iii) a density-dependence estimate, with positive values implying density-dependence with larger variation at high than at low densities, negative values indicating density-dependence with larger variation at low than at high densities, and values around zero implying weak or no density-dependence (density-dependence has an effect on population variability [67,68], although this effect might not always be large [69]); (iv) sampling effort, estimated as the number of fieldworkers performing bird censuses in a country divided by country area (a more intense sampling effort might result in some cases in a larger perceived population size [29]); (v) census method, considering whether exclusively point-counts or other methods (e.g. line transects or territory mapping) had been used (estimates of population variability are larger when exclusively point-counts are used [29]); (vi) habitat fragmentation due to urbanisation, transport infrastructure and agriculture (highly fragmented habitats are associated with high levels of population variability [29]); and (vii) number of years with population indices (variability in population size increases with the duration of the time series ([70] and references therein)). A detailed description of how these parameters were obtained or assessed can be found in [29]. The GLM had the statistics *F*_237,951_ = 4.70, adjusted *r*^2^ = 0.425, *P* < 0.0001. Least squares means of SEE from the GLM for every bird species were our estimates of population fluctuations controlled for the parameters mentioned above. These estimates of population fluctuations followed an approximately normal distribution without further transformation.

The variance linked to the precision of population size estimates (i.e., observation or measurement error) is known to bias the estimates of some population parameters [67,71,72]. In our case, observation error was unknown, but we assumed that it was similar across species and countries because standardized methodologies were used by all national bird census programmes, and when methods differed among countries, this was statistically controlled (e.g. by including the census method in the analysis; see above). In an attempt to evaluate the reliability of our estimates of population fluctuations, these were compared with the variance of the population dynamics (the so-called process variance) assessed for 35 common bird species in the UK (the pheasant *Phasianus colchicus* was excluded because it is introduced in Europe) using refined methods that take the observation error into account [30]. According to phylogenetic generalized least square regression models (these tests allowed us to control for the number of countries used to calculate population fluctuations and for similarities among species due to common ancestry; see Statistical analysis below for details), we found that both types of estimates were positively related either excluding (estimate ± SE = 0.280 ± 0.072, *t* = 3.89, *P* = 0.00046, λ = 0.000) or including (estimate ± SE = 0.303 ± 0.070, *t* = 4.33, *P* = 0.00014, λ = 0.000) body mass in the model, suggesting that our estimates of population fluctuations were indeed reliable and repeatable across methods. It should be mentioned that the two types of estimates were not expected to be identical, since they were assessed in different geographic areas (continental Europe versus UK), but when populations are sufficiently large, as is the case of these common bird species, they tend to fluctuate similarly across countries [30,73,74].

### Ecological, demographic, life-history and genetic characteristics of bird species

#### Abundance

Population size (number of breeding pairs) in the Western Palearctic west of the Ural Mountains was obtained from [75], derived in a consistent way from national bird census programmes in all countries. The arithmetic mean of the maximum and minimum estimates was used. The hypothetical association between abundance and the magnitude of population fluctuations might be spuriously affected by measurement error if measurement error varied consistently with respect to abundance. However, according to a comparison between abundance estimates from [75] and from an independent source [76] for a single country (Spain), there was no evidence for such consistent variation [35].

#### Distribution range

Western Palearctic breeding range was calculated as the area of the shape comprised by the greatest span of latitude and longitude of each species’ breeding range in the Western Palearctic, using information from distribution maps in [64]. This area was estimated with the following equation: area = RE^2^ x (longitude_1_ − longitude_2_) x (sin[latitude_1_] − sin[latitude_2_]); where RE is the radius of the Earth (6366.2 km) and latitude and longitude are expressed in radians. Total breeding range was calculated in a similar way but also including breeding areas outside the Western Palearctic. When the breeding range included parts of America, Old and New World ranges were calculated separately and subsequently summed. Ranges estimated with these methods overestimate true geographical ranges, but both are strongly positively correlated [35].

#### Heterogeneity of distribution

Heterogeneity of distribution was estimated as the coefficient of variation in population density among European countries. Population density for each country was estimated as the geometric mean from minimum and maximum population size estimates divided by area of countries in square kilometres. The geometric population size mean was calculated by the equation exp([(log_10_[minimum value + 1] + log_10_[maximum value + 1])/2] - 1) because of the exponential nature of the data. Maximum and minimum estimates of population size (number of breeding pairs) for each country were obtained from [77]. Information for countries not present in [77] was obtained from [75], either directly or using the values reported for the group “other countries” and assigning the breeding pairs proportionally to the different countries according to their area (for more details, see [35]).

#### Migration

Migration distance was estimated as the breeding latitude minus the wintering latitude, considering latitudes in the Southern Hemisphere as negative values. Breeding and wintering latitudes were determined as the mean of the northernmost and southernmost latitudes of the breeding and the wintering distribution, respectively, to the nearest tenth of a degree. Breeding and wintering ranges were obtained from maps in [64]. This method to calculate migration distance has already been used in previous studies (e.g. [27]).

#### Dispersal

Maximum dispersal distance was estimated as the minimum distance from the mainland to an island with a permanent breeding population, using information from distribution maps in [64]. When another island was located between the mainland and the island with a permanent breeding population, inter-island distance or the minimum distance from the mainland to the intermediate island (the longest one) was considered (for more details, see [78]). The estimate of maximum dispersal distance was a minimum estimate because birds travelling to islands may not have taken the shortest route.

A second estimate of dispersal was natal dispersal, i.e., the distance (in km) between the locations where birds were ringed as nestlings (or fledglings) and recovered as breeders, derived from [79]. These data were based on information recorded by the British Trust for Ornithology in the British Isles during an extended period of time (1909–1994). Geometric mean distances (for detailed information on calculations, see [79]) were used in the present study following [80].

#### Adult survival

Annual adult survival rate was obtained from [64]. If multiple estimates were provided, information from the UK was used because those estimates were generally based on the largest sample sizes.

#### Predation

Susceptibility to predation by one mammalian (domestic cat (*Felis catus*)) and two avian (goshawk (*Accipiter gentilis*) and sparrowhawk (*Accipiter nisus*)) predators was estimated as the log_10_-transformed abundance of prey minus the log_10_-transformed expected number of prey, both referred to Denmark. Prey remains of both raptors were systematically collected near their nests [81], while specimens killed by cats were brought by people to a taxidermist (J. Erritzøe). The expected number of prey was estimated as the proportion of individuals of each bird species, based on standardized point count censuses [82,83], multiplied by the total number of prey individuals. The index of prey susceptibility was positive when prey was more common (and negative when prey was less common) than expected from their abundance in the environment. This method has been used and described in detail in previous studies (e.g. [83–85]).

Flight initiation distance (FID) is an indirect estimate of susceptibility to predation, because it reflects the risk that animals are willing to take when approached by a potential predator. FID was assessed by one of us (APM) during 2006–2008 using a standardized technique. Briefly, when a bird had been located with binoculars, the observer moved at a normal walking speed towards the bird, and the distance (in meters) at which it took flight was recorded as FID. If the bird was positioned in the vegetation, the height above ground was recorded to the nearest metre, and FID was estimated as the square root of the sum of the squared horizontal distance and the squared height above ground level [86]. The distance at which the observer started walking towards the bird when first observed was at least 30 m and relatively constant across species [87]. For further details on the methods and reliability of FID estimates across spatial and temporal scales, see [88–90].

Nest predation rate was obtained from [64] and [91] combined with data from the primary literature. If multiple estimates were available, we used the mean estimate weighted by sample size.

#### Parasitism

The level of parasitisation was estimated as the number of blood parasite species found in the bird species according to [92] and [93], combined with information from several sources listed in [94].

#### Reproductive rate

Clutch size (mean number of eggs) and maximum number of clutches per season were obtained from [64]. If multiple estimates were provided, information from the UK was used because those estimates were generally based on the largest sample sizes. Annual fecundity was calculated by multiplying clutch size by maximum number of clutches per season.

#### Body size

Mean body mass of males and females was obtained from [64]. If multiple estimates were provided, information from the UK was used because those estimates were generally based on the largest sample sizes. Body mass was calculated as the mean of male and female body mass.

#### Brain size

Relative brain mass was estimated as the residuals after regressing log_10_-transformed brain mass on log_10_-transformed body mass while simultaneously controlling for phylogenetic relationships among species (see Statistical analysis below for details). Brain size was obtained from an extensive database recorded by a taxidermist (J. Erritzøe) on post-mortem examination of dead birds collected in Denmark. Brains were weighed to the nearest milligram, always excluding damaged heads. Further information can be found elsewhere [87,95,96].

#### Sexual dichromatism

Bird species were classified as sexually dichromatic or monochromatic depending on whether males and females were readily distinguishable based on plumage coloration according to field guides [97,98]. Monochromatic species were given a score of zero and dichromatic species a score of one. This classification restricts sexual dichromatism to the visible domain for humans, but measures of coloration derived from models based on avian vision are strongly positively related [99,100]. The same dichotomous categorization of sexual dichromatism has already been used in previous studies [81,84,94].

#### Coloniality

Bird species were classified as colonial or non-colonial (solitary) depending on whether individuals breed in densely distributed nesting territories that contain no resources other than nesting sites or in multi-purpose territories. Information was obtained from [64].

#### Habitat

Bird species were classified as terrestrial (0; not commonly encountering water), partly aquatic (1; spending at least part of the time in water), or completely aquatic (2; spending most or all of the time in water) based on habitat descriptions in [64].

#### Number of subspecies

Total number of subspecies in the entire breeding range was recorded using [64] as a source.

#### Genetic variability

Different estimates of genetic variability were obtained. The band-sharing coefficient is an estimate of the number of shared minisatellite bands in relation to the total number of bands among adults [101]. A high band-sharing coefficient implies that the genetic variability in the population is relatively low. Information on band-sharing coefficients derived from an extensive search of the literature (references can be found in [102]).

We obtained information on genetic variability for microsatellites in birds by an exhaustive search of Web of Science using the terms microsat*, micro-sat* and bird*. The number of alleles, the proportion of polymorphic loci, observed (H_o_) and expected heterozygosity (H_e_) and sample size were extracted. If more than one estimate was available, we calculated mean estimates weighted by sample size. The inbreeding coefficient *F* was calculated as *F* = 1 − H_o_ / H_e_ [103].

#### Advancement of spring migration phenology

Information on advancement in mean arrival date and first arrival date during spring migration estimated as the change in arrival date per year was obtained from the literature and from E. Lehikoinen (pers. comm.). These two parameters have been used in previous studies (e.g. [27,62,104,105]), although very often with different specific values due to the continuous update of the information.

#### Colonisation of urban areas

A species was considered as urbanised if breeding populations occur inside towns and cities and population densities in towns and cities are higher than in rural habitats [63]. Information on urbanisation was obtained from [64] and [91].

The search for information on ecological, demographic, life-history and genetic parameters was finished by 15^th^ July 2009. Data for all bird species included in the study are reported in S1 Table (Supporting information).

### Statistical analysis

Some of the species-specific characteristics had to be transformed before further analysis to approach a normal distribution. The following transformations were performed (variables in brackets): log_10_(abundance, heterogeneity of distribution, natal dispersal, FID, clutch size, body mass, number of subspecies, band-sharing coefficient, and number of alleles), log_10_(distance to mainland + 1), (Western Palearctic range)^2^, (inbreeding coefficient + 0.5)^2^, (annual fecundity)^0.2^, sqrt(total breeding range, adult survival, migration distance, and nest predation), and arcsin(sqrt(polymorphic loci)). In all cases, we chose the transformation that was more successful in approaching the frequency distribution of that variable to a normal distribution. All transformations were chosen blind with regard to the effect of transformation on the results. After transformation but before the analyses, Western Palearctic range and total breeding range were divided by 100 and 1000, respectively, to produce variables with a similar scale.

Possible relationships between the magnitude of population fluctuations and species-specific parameters were tested with phylogenetic generalized least square regression models [106–108] implemented in R statistical environment [109]. We used function pglm3.3 developed by R. P. Freckleton (University of Sheffield, UK), libraries ‘‘MASS”, ‘‘mvtnorm” and ‘‘ape”, and the avian phylogeny reported in [110]. The method applies a maximum likelihood modelling approach to estimate the phylogenetically corrected partial correlation between the variables of interest [111]. The optimum degree of phylogenetic dependence was identified for each model, and the corresponding lambda parameter (λ) included in subsequent analyses. Our estimates of population fluctuations were derived from a different number of populations (countries) for each bird species (range = 1–12; [29]), and this implies that different species did not provide equally precise information on this parameter. Equal treatment of every species in the analyses would be inappropriate [112] and, consequently, weighted analyses were performed using the number of countries from which population fluctuations had been estimated as a weight. Specifically, a matrix of 1/weights was added as an error term, and this term was multiplied by different values until the value providing the highest maximum likelihood was found. This method has been used and described in detail in previous studies (e.g. [113]). Not only phylogenetic relations among species, but also the number of populations used to estimate population fluctuations were controlled in all analyses including population fluctuations. Within-species heterogeneity in population fluctuations was not directly considered in the analyses, and it is known that neglecting intraspecific variability in comparative studies may cause bias [112]. However, intraspecific variability in population fluctuations was indirectly controlled, because the standard error of our estimates of population fluctuations at the species level was strongly negatively related to the number of populations (estimate ± SE = -0.0067 ± 0.0002, *t* = -31.79, *P* < 0.0001, adjusted *r*^2^ = 0.815, λ = 0.000), and the number of populations was indeed controlled in the analyses.

Number of species with information for each parameter ranged from only 35 in the case of inbreeding to all 231 species in the case of sexual dichromatism, coloniality, habitat or colonisation of urban areas (Table 1). To avoid a drastic reduction in sample size (and thus statistical power) for many of the parameters, we decided not to include all parameters simultaneously in the same model. We instead analysed all species-specific parameters one by one as independent variables, with our estimate of population fluctuations as the dependent variable. When the analysis provided a *P*-value less than 0.10, the test was repeated, but also including in the model the parameter most strongly related to fluctuations (i.e., abundance; see Results) as a confounding variable. Finally, all parameters significantly related to our estimate of population fluctuations in these latter analyses were included simultaneously in the same model.

**Table 1:** Relationships between the magnitude of population fluctuations (response variable) and a number of ecological, demographic, life-history and genetic parameters of European breeding bird species according to phylogenetic generalized least square regression models.

Parameter	Including population trend				Also including abundance			
	Estimate (SE)	*t*	*N*	λ	Estimate (SE)	*t*	*N*	λ
Dichromatism	-0.020 (0.015)	-1.35	231	0.530				
Coloniality	0.063 (0.017)	3.70***	231	0.356	0.053 (0.015)	3.62***	227	0.000
Water habitat	0.049 (0.011)	4.34***	231	0.473	0.023 (0.010)	2.20*	227	0.402
Urbanisation	-0.053 (0.013)	-3.91***	231	0.694	-0.001 (0.013)	-0.07	227	0.000
Body mass	0.006 (0.015)	0.38	229	0.588				
Clutch size	0.041 (0.045)	0.91	229	0.531				
Annual fecundity	-0.012 (0.044)	-0.29	228	0.517				
Abundance	-0.064 (0.006)	-10.35***	227	0.000				
Density ^a^	-0.074 (0.008)	-8.81***	227	0.416				
W. Palearctic range	-0.003 (0.005)	-0.60	227	0.550				
Total range	0.009 (0.003)	3.00**	227	0.407	0.011 (0.002)	4.53***	227	0.000
No. subspecies ^b^	-0.045 (0.019)	-2.40*	227	0.336	-0.012 (0.016)	-0.77	227	0.352
Migration	-0.004 (0.003)	-1.35	205	0.470				
Parasitism	-0.007 (0.003)	-2.42*	189	0.469	0.002 (0.003)	0.76	187	0.000
Relative brain mass ^c^	0.046 (0.063)	0.72	187	0.459				
Distance to mainland	-0.022 (0.014)	-1.62	153	0.658				
Survival rate	-0.012 (0.008)	-1.54	143	0.529				
Flight initiation dist.	0.017 (0.029)	0.58	140	0.656				
Heterogeneity distrib.	0.108 (0.037)	2.95**	139	0.692	-0.038 (0.040)	-0.95	138	0.556
First arrival date	-0.040 (0.025)	-1.57	139	0.305				
Mean arrival date	0.028 (0.034)	0.82	96	0.160				
Nest predation	-0.003 (0.006)	-0.54	85	0.182				
Sparrowhawk ^d^	0.012 (0.011)	1.08	82	0.595				
Goshawk ^d^	0.009 (0.012)	0.77	76	0.000				
Natal dispersal	0.072 (0.020)	3.62***	69	0.000	0.008 (0.021)	0.39	69	0.488
Cat ^d^	0.009 (0.017)	0.56	55	0.000				
Band-sharing coef.	0.027 (0.083)	0.33	49	0.764				
Alleles	-0.087 (0.062)	-1.40	40	0.000				
Polymorphic loci	-0.208 (0.102)	-2.03*	39	0.000	-0.134 (0.095)	-1.41	39	0.000
Inbreeding coef.	0.001 (0.130)	0.01	35	0.000				

Species population trend was included in all models. Phylogenetic relations among species and the number of populations used to estimate population fluctuations in each species were controlled in all analyses (see Statistical analysis for details). Lambda parameter (λ), the optimum degree of phylogenetic dependence, is shown for each model. When the relationship between population fluctuations and another parameter had a *P*-value less than 0.10, the test was repeated including also abundance in the model (second column). Abundance was always statistically significant (|estimate| ≥ 0.044, |*t*| ≥ 2.96, *P* ≤ 0.0055) in models of the second column.

* *P* < 0.05,

** *P* < 0.01,

*** *P* < 0.001.

^a^ Abundance and Western Palearctic breeding range were included in the model.

^b^ Total breeding range was also included in the model.

^c^ Brain mass controlled for body mass (residuals after regressing log_10_-transformed brain mass on log_10_-transformed body mass while controlling for phylogenetic relations among species).

^d^ Body mass and (body mass)^2^ were also included in the model because predators usually have an optimal prey size.

The way in which the degree of population fluctuations was calculated (i.e., dispersion around the trend; see Introduction) provided estimates independent of population trends. However, our estimates of population fluctuations at the species level might still be related to population trends for other reasons [114], so we checked whether this was the case. First, population indices were regressed on years for every population and the slope of the regression line was considered to represent the population trend [29]. We then performed a GLM with the slope from every regression (i.e., from every population) as the dependent variable, species as a fixed factor, and the parameters presumably affecting temporal variability in population size (marginality, latitude, density-dependence, sampling effort, census method, habitat fragmentation, and number of years; see Estimates of population fluctuations in bird species) as independent variables. The GLM had the statistics *F*_237,951_ = 2.02, adjusted *r*^2^ = 0.170, *P* < 0.0001. Least squares means of slopes from the GLM for every bird species were our estimates of population trend statistically controlled for the parameters mentioned above. These estimates of population trend were log_10_(x + 0.14)-transformed before further analysis to approach a normal distribution. We found that population fluctuations and population trends were marginally non-significantly related (estimate ± SE = 0.055 ± 0.029, *N* = 231, *t* = 1.89, *P* = 0.061, λ = 0.541) in an analysis that also controlled for the number of countries used to calculate population parameters and for similarities among species due to common ancestry (see above). As the relationship between population fluctuations and population trend was close to statistical significance, and because we were particularly interested in studying the relationship between population fluctuations and other species-specific parameters independent of population trend, we included population trend as a covariate in all subsequent analyses on population fluctuations.

Possible relationships between species-specific parameters other than population fluctuations or population trends (e.g. between abundance and urbanization, or between number of subspecies and natal dispersal) were also tested with phylogenetic generalized least square regression models, i.e., controlling for phylogenetic relationships among species (see above), but without controlling for the number of countries used to calculate population fluctuations.

## Results

**Fig 1:**
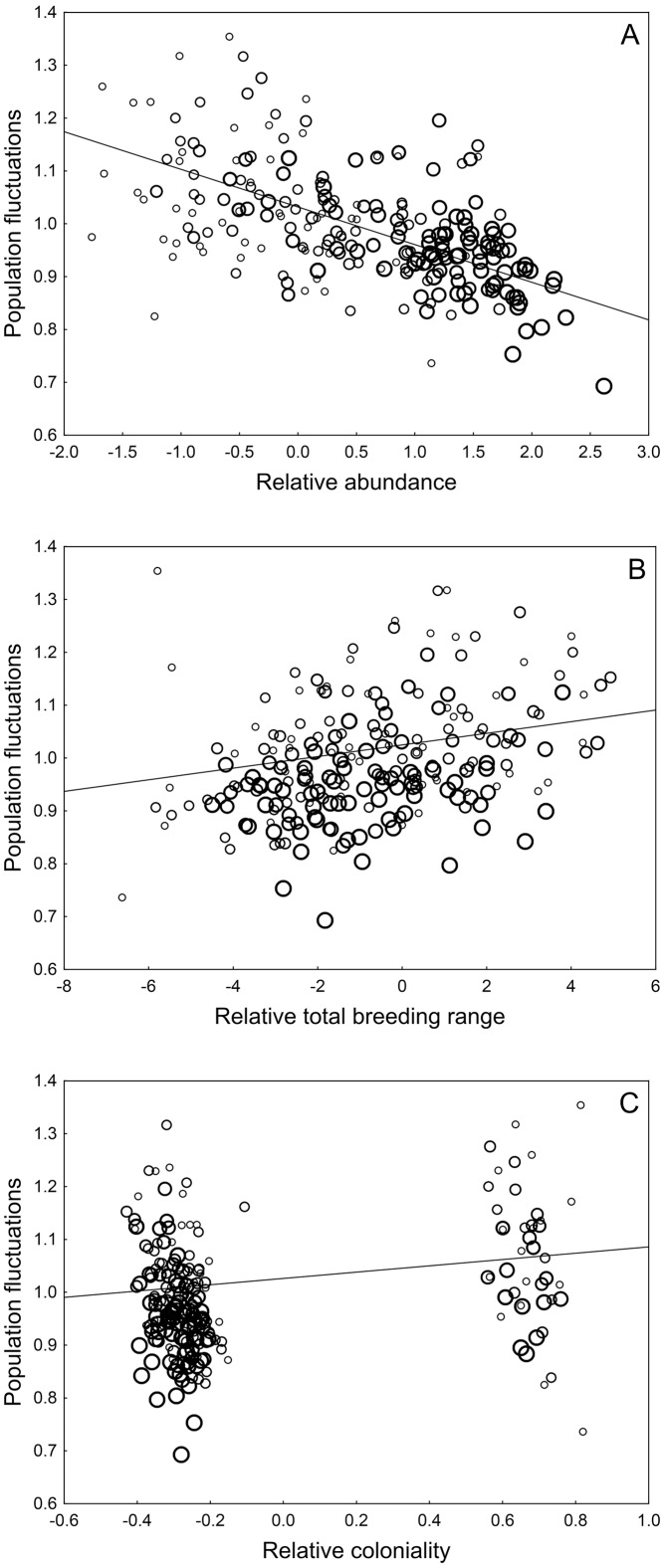
Relationships between the estimates of population fluctuations and (A) relative abundance, (B) relative breeding range, and (C) relative coloniality in European breeding bird species. Relative abundance was estimated as the residuals from a model with abundance as the response variable and total breeding range, coloniality and population trend as predictors. Relative breeding range was estimated as the residuals from a model with total breeding range as the response variable and abundance, coloniality and population trend as predictors. Relative coloniality was estimated as the residuals from a model with coloniality as the response variable and abundance, total breeding range and population trend as predictors. All variables except coloniality and population fluctuations were transformed before the analyses (see Statistical analysis). Lines are best-fit regressions (a: y = 1.032–0.071 x; b: y = 1.025 + 0.011 x; c: y = 1.026 + 0.060 x). All models and regressions took into account the number of countries used to calculate population trends and fluctuations (bubble size indicates this number; range = 1–12) and similarities among species due to common ancestry (see Statistical analysis for details).

When species-specific parameters were analysed one at a time, the magnitude of population fluctuations was positively related to coloniality, water habitat, total breeding range, heterogeneity of distribution and natal dispersal, and negatively related to urbanisation, abundance, relative number of subspecies, parasitism and proportion of polymorphic loci (Table 1). When both Paleartic breeding area and abundance were included in the model (which is equal to inclusion of population density), population fluctuations were still negatively related to abundance, implying that populations fluctuated less not only with large population size but also with high density. All other species-specific characteristics here were not significantly related to population fluctuations (Table 1). Abundance (either with or without Palearctic area included in the model) was the parameter most strongly related to population fluctuations (Table 1). When abundance was included in models showing significant relationships between population fluctuations and other variables, most of these relationships were not statistically significant, but coloniality, water habitat and total breeding range remained positively and significantly related to population fluctuations (Table 1). Abundance was negatively and significantly related to population fluctuations in all cases (Table 1). The marginally non-significant relationship between population fluctuations and population trends (see Statistical analysis) was positive and marginally significant (estimate ± SE = 0.055 ± 0.027, *N* = 227, *t* = 2.00, *P* = 0.047, λ = 0.000) when both population trend and abundance were included in the same model. When abundance, total breeding range, coloniality, water habitat and population trend were included simultaneously in the same model, all these parameters except water habitat were significantly related to population fluctuations (Table 2, Fig 1). When water habitat was excluded from the model, all other parameters remained significantly related to population fluctuations (S2 Table (Supporting information)).

**Table 2:** Relationships between the magnitude of population fluctuations (response variable) and abundance, total breeding range, coloniality, water habitat and population trend of European breeding bird species according to a phylogenetic generalized least square regression model.

Term	Estimate (SE)	*t*	*P*
Intercept	1.157 (0.047)	24.73	< 0.0001
Abundance	-0.065 (0.008)	-7.98	< 0.0001
Total range	0.009 (0.002)	3.77	0.00021
Coloniality	0.039 (0.015)	2.57	0.011
Water habitat	0.010 (0.010)	0.92	0.36
Population trend	0.052 (0.026)	2.00	0.047

Phylogenetic relations among species and the number of populations used to estimate population fluctuations in each species were controlled in the analysis (see Statistical analysis for details). The model had the statistics: *F* = 22.67, adj-*r*^2^ = 0.324, *N* = 227, *P* < 0.0001, λ = 0.438.

Most parameters significantly related to the degree of population fluctuations when analysed one by one were also significantly related to abundance, with the exception of coloniality, relative number of subspecies and proportion of polymorphic loci (Table 3). As the number of subspecies was predicted to be related to population fluctuations because of its relationship with dispersal ([55]; see Introduction), the relationships between the number of subspecies (controlled for total breeding range) and the two estimates of dispersal (distance to mainland and natal dispersal) were also tested. Indeed, we found that the number of subspecies was positively related to distance to mainland (estimate ± SE = 0.111 ± 0.052, *N* = 153, *t* = 2.16, *P* = 0.033, λ = 0.805), and negatively related to natal dispersal distance (estimate ± SE = -0.274 ± 0.075, *N* = 69, *t* = -3.65, *P* = 0.00052, λ = 0.629). We also found that Western Palearctic breeding range was negatively related to the proportion of polymorphic loci (estimate ± SE = -4.199 ± 1.682, *N* = 39, *t* = -2.50, *P* = 0.017, λ = 0.000), coloniality was positively related to water habitat (estimate ± SE = 0.166 ± 0.053, *N* = 231, *t* = 3.14, *P* = 0.0019, λ = 0.832) and both Western Palearctic breeding range and distance to mainland were significantly related to abundance (Table 3).

**Table 3:** Relationships between abundance (response variable) and a number of ecological, demographic, life history and genetic parameters of European breeding bird species according to phylogenetic generalized least square regression models.

Parameter	Estimate (SE)	*t*	*N*	λ
Coloniality	0.006 (0.134)	0.04	227	0.752
Water habitat	-0.386 (0.096)	-4.01***	227	0.692
Urbanisation	0.756 (0.097)	7.79***	227	0.811
W. Palearctic range	0.209 (0.029)	7.28***	227	0.780
Total range	0.070 (0.020)	3.53***	227	0.797
No. subspecies ^a^	0.232 (0.140)	1.66	227	0.774
Parasitism	0.170 (0.022)	7.70***	187	0.799
Distance to mainland	0.415 (0.100)	4.17***	153	0.748
Heterogeneity distrib.	-1.952 (0.235)	-8.31***	138	0.685
Natal dispersal	-0.608 (0.155)	-3.93***	69	0.819
Polymorphic loci	0.343 (1.225)	0.28	39	0.705

Phylogenetic relations among species were controlled in the analyses (see Statistical analysis for details).

*** *P* < 0.001.

^a^ Total breeding range was also included in the model.

## Discussion

The magnitude of population fluctuations of European bird species was related to a number of species-specific ecological, demographic, life-history and genetic characteristics independent of population trends. However, some of these relationships were non-significant with small effects when abundance (breeding population size) was controlled in the analyses. In fact, some of these species characteristics were predicted to be related to population fluctuations because of their association with abundance (e.g. heterogeneity of distribution or colonisation of urban areas; see references in Introduction), so it is not surprising that they were no longer related to population fluctuations when also abundance was considered in the statistical models. Most species parameters significantly related to population fluctuations when analysed one at a time, but non-significantly related when including abundance in the model, were indeed also related to abundance, with the relative number of subspecies and the proportion of polymorphic loci being the only exceptions. Although a direct link between these two parameters and abundance could not be stablished, an indirect link was found. Namely, the relative number of subspecies was related to the two estimates of dispersal (natal dispersal and distance to mainland), and, in turn, these two estimates of dispersal were also related to abundance. In addition, the proportion of polymorphic loci was related to Western Palearctic breeding range, a parameter that was also related to abundance. These indirect links between number of subspecies or proportion of polymorphic loci on one side and abundance on the other might help explain why the relationships between population fluctuations and these two parameters were no longer significant when abundance was included in the models. Finally, the positive and significant relationship between coloniality and water habitat might explain why the relationship between population fluctuations and water habitat was no longer significant when both coloniality and water habitat were included in the same model.

Coloniality, total breeding range and abundance were the only parameters that predicted the magnitude of population fluctuations of European breeding bird species when a number of confounding factors such as other species parameters, population trends, phylogenetic relationships among species, and number of countries used to estimate population fluctuations were taken into account. The positive relationship between population fluctuations and total breeding range was an unexpected result, because abundance is generally positively related to total breeding range ([32,33], this study), but negatively related to population fluctuations ([30], this study). The fact that a positive instead of a negative relationship was found suggests that the association between population fluctuations and total breeding range is not mediated by population size. In agreement with this suggestion, the magnitude of population fluctuations was positively and significantly related to total breeding range regardless of whether abundace was controlled in the analysis. We can speculate that larger breeding ranges probably imply a wider range of environmental conditions, and, therefore, greater environmental stochasticity which, in turn, will result in wider population fluctuations [31]. Alternatively, larger breeding ranges might lead to a more pronounced population structuring, i.e., to the formation of more subpopulations which are more distant from each other, implying a lower relative migration rate among the full set of subpopulations. In turn, lower levels of migration will increase the amplitude of fluctuations within subpopulations [115], although asynchrony in fluctuations among subpopulations will make the entire metapopulation more stable [115]. It should be noted that in this study we were dealing with fluctuations within each country, and country-level populations might be considered subpopulations with respect to the entire European population.

Regarding coloniality, colonial species fluctuated more than solitary ones, the expected result if coloniality is associated with patchy and unpredictable environments, i.e., with environmental stochasticity [56]. Although different hypotheses for the evolution of coloniality have been proposed (review in [116]), many scientists suggest that the primary adaptive function of coloniality is to enhance foraging efficiency when food is clumped and/or unpredictable. For example, indivuals might benefit from the searching abilities of successful individuals if these are followed away from the colony on foraging trips (information centre hypothesis [117,118]), or might benefit from group foraging (recruitment centre hypothesis [119,120]). The two above mentioned hypotheses assume that coloniality emerged for a more efficient exploitation of patchy and/or unpredictable resources. However, the reverse causal relationship is also possible: coloniality might have emerged for reasons unrelated to foraging efficiency, but once it evolved, coloniality made the exploitation of patchy and/or unpredictable resources possible. This latter scenario seems to be the most plausible explanation for the strong association between coloniality and marine habitat (characterized by unpredictable, patchy and ephemeral food resources) in birds [56]. The fact that populations fluctuate more widely in colonial than in non-colonial species suggests a previously unrecognized cost of coloniality, given that wide fluctuations increase the risk of extinction [6,121]. Moreover, coloniality very probably entails some degree of positive density-dependence because, otherwise, solitary breeding would spread and become prominent in the population. In turn, positive density-dependence (the so-called Allee effect) generally destabilizes population dynamics and makes populations more vulnerable to extinction [122]. However, this possible negative effect of coloniality on population viability apparently contradicts previous studies in which solitarily breeding bird species showed more pronounced population declines than colonial ones [123]. Clearly, more research is needed to understand the possible role of coloniality in population persistence.

Abundance was the species-specific parameter most strongly related to population fluctuations, with population size fluctuating more in less abundant species. As the relationship between population fluctuations and population size was qualitatively identical when Palearctic breeding area was included in the statistical model, the same result applies to density, i.e., population size fluctuated more in species with less dense populations. The relationship between mean and temporal variability in population size has been studied for decades (e.g. [30,124–128]), with a general pattern arising: temporal variability in population size (measured as standard deviation or variance among years) increases with abundance, while the slope of the regression between both (logarithmically transformed) parameters is generally less than one [128]. In other words, the relative increase in variability is smaller when populations become more abundant. This implies that when temporal variability is measured as the coefficient of variation (standard deviation divided by the mean), the relationship between variability and abundance becomes negative [128]. A negative relationship between temporal variability and abundance was also found when variability was calculated with more refined methods that included both demographic and environmental variances [30]. As illustrated by these two studies [30,128], a negative relationship between temporal variability in population size and abundance has previously been suggested. The novelty of the present study is that the relationship between population fluctuations and population size was still negative and significant when the potentially confounding effect of population trend was controlled. Small population size, regardless of population trend, was a key factor associated with wide population fluctuations. Taking population trends into account might be important when studying the relationships between population size and fluctuations, because population trends and fluctuations are often positively related ([114], this study).

We assumed a similar measurement error in our estimates of population size across species (see Methods), but measurement error might still play a role in the negative relationship between population fluctuations and abundance. For mathematical reasons, population size estimates might contain more noise relative to its size in rare than in abundant species, even if absolute measurement error was similar for all species. However, it should be noted that only data from the Pan-European Common Bird Monitoring Scheme were used, so rare species could not bias our results simply because they were not included in the study. Obviously, among the common bird species, some are more abundant than others, but the smaller the range of abundance across species, the less probable it is that measurement error is strongly affecting our results. An argument against an association between population size and measurement error comes from previous studies [29] that included exactly the same population indices (i.e., same countries, species and years) used in the present study. When the coefficient of variation (CV) of population indices was calculated separately for years with high and low population indices within populations, CV-high (temporal variation in population size when abundance was high) and CV-low (temporal variation in population size when abundance was low) did not differ significantly from each other [29]. If relative measurement error increased as abundance decreased, CV-high should be smaller than CV-low, but that was not the case. Therefore, although we cannot completely rule out the possibility that measurement error is contributing to some extent to the relationship between population fluctuations and population size, the arguments shown above suggest that such a contribution, if present, is probably small in our case, and was not the cause (or not the only cause) for the strong negative relationship we found. A further limitation of the unavailability of estimates of measurement error for population indices was that this error could not be carried forward in subsequent analyses.

Three types of non-mutually exclusive stochastic processes might explain the negative relationship between population size and fluctuations. First, demographic stochasticity (random variation in fitness among individuals) will have a larger effect on population dynamics when population size is small [31]. Second, environmental stochasticity (random fluctuations in environmental conditions) will have a reduced effect in large populations because these populations generally cover large geographic areas [32,33], and asynchrony in fluctuations among distant subpopulations will make the entire population more stable [115]. However, it has also been suggested that the effects of environmental stochasticity on long-term population dynamics will be independent of population size [31]. Third, genetic stochasticity (random genetic drift and inbreeding depression) will reduce population mean fitness [61] and, consequently, population size. All these stochastic processes imply a causal relationship between population size and fluctuations, mostly with small population sizes causing large fluctuations. Specifically, small population size would enhance the effects of different types of stochasticity on population dynamics, thus increasing temporal variability in population size. Causality might also work in the opposite direction, with population fluctuations leading to a reduction in population size. For example, the bottlenecks associated with wide fluctuations will cause a reduction in genetic variability [58,59], which, in turn, will have a negative effect on population mean fitness and size [60,61]. However, the relationship between population size and fluctuations does not necessarily involve causation. Some bird species, for example, show a source-sink dynamic among subpopulations, with large population size and small fluctuations in the centre of the distribution range and the opposite in the periphery [127]. In this case, the relationship between abundance and fluctuations might be mediated by a third parameter, the geographic position within the range.

It has been repeatedly suggested that the time to extinction of populations depends on population size (e.g. [30,31,129,130]). This process might be mediated, at least partially, by the relationship between abundance and the degree of population fluctuations, because smaller populations fluctuate more widely than larger ones ([30], this study) and wide fluctuations increase the risk of extinction [6,121]. For a given mean abundance (and other things being equal), larger fluctuations imply that population size comes closer to zero in the low phases of fluctuations, thus increasing the risk of extinction. Moreover, as mentioned above, the dramatic reduction in population size in the low phases of wide fluctuations entails a reduction in genetic variability [58,59]. This is the result of different interacting processes that involve random genetic drift and inbreeding. On the one hand, alleles that pass from one generation to the next change their frequencies simply by chance, and the probability of some alleles becoming fixed and others lost increases in small populations [131]. On the other hand, the probability of mating with a relative (thus producing inbred offspring) and the rate at which inbreeding accumulates over generations also increase in small populations [131]. Whatever mechanism is responsible for the reduction in genetic variability associated with small population size, this reduction inevitably diminishes the capacity of species to adapt to environmental changes, thus increasing the probability of extinction [132]. Overall, small population size, wide fluctuations, demographic and environmental stochasticity, and loss of genetic diversity seem to reinforce one another, leading to an acceleration of the extinction process [133].

Bird populations fluctuated less in abundant species and also in species declining in numbers, findings that suggest a link between abundance and population trend. This link was indeed documented for European bird species in the last decades of the 20^th^ century [134], a period that roughly corresponds with the current study [29]. During the late 20^th^ century, bird abundance decreased dramatically in Europe, but this reduction in abundance mostly affected common (small) species, while rare (large) ones remained relatively stable or even increased in population size [134]. Our study shows that these common species that suffered a reduction in numbers were the ones whose populations fluctuated the least, i.e., they were steadily declining. As these common species are now less abundant, and abundance is negatively associated with fluctuations, we may infer that they are now fluctuating more widely than in the past, with possible implications for conservation (see references in Introduction). In any case, the declining trends in common European birds in the late 20^th^ century have become stabilized at the beginning of the 21^st^ century, with still relatively high population sizes [134].

Previous studies on a wide range of organisms, including plants, fish or mammals, found that species with a slow pace of life (i.e., species with long life span, late maturity, long generation times, and low fecundity) showed in general smaller temporal variability in population size and other demographic traits than fast-living species [135–138]. Similarly, previous studies on European bird species found that the magnitude of population fluctuations was related to life-history or ecological traits, such as migration [36], clutch size, or survival [30]. Specifically, resident species with large clutch size and low survival fluctuated more widely than migratory low-reproducing long-lived species. However, in the present study, none of these life-history or ecological factors predicted interspecific variability in population fluctuations. We can speculate about at least four possible reasons for the different results found in this and previous studies on European birds. First, we were particularly careful in removing any possible effect of population trend on the relationships between population fluctuations and species-specific characteristics. In contrast, previous studies did not include population trend (or population growth rate) in statistical models testing for such relationships. Second, our study included data from 12 European countries covering a relatively wide range of latitudes and longitudes (from Spain to Finland), while previous studies dealt with population fluctuations in one country only (Czech Republic [36] or UK [30]). Population dynamics might differ at different spatial scales (e.g. national versus continental) because avian communities do not respond to environmental variation in a similar fashion across regions [139], or because asynchrony among populations increases with distance [140]. Nevertheless, the positive relationship between our estimates of population fluctuations and those found in a single country (UK; see Estimates of population fluctuations in bird species in Methods) suggests that similar fluctuations occurred at different spatial scales. Third, a composite of information from different countries probably added noise to our data compared to information obtained from a single country, because some factors (e.g. census methods, bird species, or number of years with information) varied among countries [29]. Although most of this variation was statistically controlled in the analyses, the possibility that some variation remained uncontrolled cannot be dismissed. In any case, the possible noise in our data did not preclude finding a strong relationship between population fluctuations and coloniality, breeding range or abundance, so we assume that any other trait strongly related to population fluctuations would have also been detected. Fourth, previous studies pointed out that the relationships between life-history traits and population dynamical patterns may be apparent only on a scale of generations, but not on a scale of years [141]. In birds, short-lived species show generation times quite close to one year, while long-lived species show much longer generation times. Therefore, studies based on a scale of years but including predominantly short-lived species might find significant relationships that are not evident when a larger proportion of long-lived species are included.

In conclusion, this study shows that in European birds, coloniality, total breeding range, and population size strongly predict the magnitude of population fluctuations independent of population trend, with colonial, more widely distributed and smaller populations fluctuating more. To our knowledge, coloniality and breeding distribution had never been suggested to be related to population fluctuations in birds. Previous findings on the negative relationship between abundance and fluctuations in bird populations are now extended to a larger number of bird species and to a larger geographical range. This study does not support previously suggested relationships between population fluctuations and migration, clutch size, or survival. The close association between population size and fluctuations suggests that population stabilization may be improved by increasing population size.

## Supporting information

S1 Table Information on ecological, demographic, life-history and genetic characteristics of European bird species. (DOCX)

S2 Table Relationships between the magnitude of population fluctuations and abundance, total breeding range, coloniality and population trend of European breeding bird species. (DOCX)
