# Supplementary material for: Colonial, more widely distributed and less abundant bird species undergo wider population fluctuations independent of their population trend
Source: PLoS One. 2017 Mar 2;12(3):e0173220. doi: 10.1371/journal.pone.0173220 (PMC5333898; doi:10.1371/journal.pone.0173220)
Supplement: S1 Table — (DOCX) [file pone.0173220.s001.docx]

**S1 Table.** Information on ecological, demographic, life-history and genetic characteristics of European bird species. Sexual dichromatism: 0 - monochromatic, 1- dichromatic. Coloniality: 0 - non-colonial, 1 - colonial. Water habitat: 0 - terrestrial, 1 - partly aquatic, 2 - aquatic. Urbanisation: 0 - not urbanised, 1 - urbanised. For information on how the different characteristics were obtained or calculated, see section Ecological, demographic, life-history and genetic characteristics of bird species in Methods.

| Species | Population fluctuations | Population trend | Sexual dichromatism | Coloniality | Water habitat | Urbanisation | Body mass (g) | Clutch size | Annual fecundity | Western Palearctic  population size | Western Palearctic  breeding range (km^2^) | Total breeding range  (km^2^) | Number of subspecies | Migration distance  (degrees) | Number of blood  parasite species | Brain mass (g) |
| --- | --- | --- | --- | --- | --- | --- | --- | --- | --- | --- | --- | --- | --- | --- | --- | --- |
| *Acanthis flammea* | 1.0409 | 0.0046 | 1 | 0 | 0 | 1 | 13.05 | 5.00 | 10.00 | 13900000 | 13525065.50 | 38772112.30 | 3 | 9.46 | 3 | 0.55 |
| *Accipiter gentilis* | 0.9870 | -0.0366 | 1 | 0 | 0 | 0 | 1139.50 | 3.64 | 3.64 | 185000 | 18056769.20 | 85849274.60 | 9 | - | 3 | 7.56 |
| *Accipiter nisus* | 1.0950 | 0.0339 | 1 | 0 | 0 | 0 | 204.00 | 4.90 | 4.90 | 395000 | 20206620.10 | 59603419.00 | 6 | 12.79 | 3 | 2.89 |
| *Acrocephalus arundinaceus* | 1.0330 | 0.0136 | 0 | 0 | 1 | 0 | 30.35 | 4.80 | 9.60 | 2200000 | 15323429.70 | 72532660.50 | 4 | 38.69 | 0 | - |
| *Acrocephalus palustris* | 0.9501 | 0.0072 | 0 | 0 | 1 | 0 | 12.00 | 4.30 | 4.30 | 5000000 | 12986579.70 | 15087288.20 | 1 | 66.84 | 1 | 0.52 |
| *Acrocephalus schoenobaenus* | 0.9818 | 0.0066 | 0 | 0 | 1 | 0 | 11.90 | 4.98 | 9.96 | 5900000 | 16946488.00 | 22251413.00 | 1 | 62.10 | 4 | 0.44 |
| *Acrocephalus scirpaceus* | 0.9324 | 0.0059 | 0 | 0 | 1 | 0 | 11.80 | 3.89 | 3.89 | 3850000 | 16341654.30 | 28303507.40 | 2 | 44.60 | 4 | 0.58 |
| *Actitis hypoleucos* | 0.9450 | 0.0021 | 0 | 0 | 1 | 0 | 47.75 | 4.00 | 4.00 | 1160000 | 18567443.20 | 56601322.70 | 1 | 44.39 | 2 | 0.78 |
| *Aegithalos caudatus* | 1.0305 | 0.0139 | 0 | 0 | 0 | 1 | 8.80 | 9.40 | 9.40 | 8500000 | 18276019.50 | 58625882.90 | 17 | 0.00 | 0 | 0.42 |
| *Alauda arvensis* | 0.8042 | -0.0144 | 0 | 0 | 0 | 0 | 36.40 | 3.69 | 14.76 | 60000000 | 18626661.40 | 52936590.50 | 15 | 13.02 | 2 | 0.90 |
| *Alcedo atthis* | 1.1527 | 0.1043 | 1 | 0 | 2 | 0 | 32.42 | 6.70 | 26.80 | 119500 | 14915965.10 | 129925253.00 | 8 | - | 2 | 0.80 |
| *Alectoris rufa* | 0.9070 | 0.0123 | 1 | 0 | 0 | 0 | 477.50 | 12.71 | 25.40 | 3250000 | 2543626.03 | 2543626.03 | 3 | - | 3 | 1.95 |
| *Anas clypeata* | 0.9359 | -0.0463 | 1 | 0 | 2 | 0 | 608.75 | 9.20 | 9.20 | 190000 | 19083579.20 | 69906542.50 | 1 | 24.07 | 2 | 3.30 |
| *Anas crecca* | 1.0443 | 0.0005 | 1 | 0 | 2 | 0 | 286.50 | 9.51 | 9.50 | 1060000 | 21256084.30 | 80853819.00 | 3 | 20.84 | 4 | 2.64 |
| *Anas platyrhynchos* | 1.0167 | 0.0128 | 1 | 0 | 2 | 1 | 1119.00 | 9.59 | 9.60 | 4200000 | 22392575.30 | 118317547.00 | 7 | 8.13 | 3 | 5.00 |
| *Anas querquedula* | 0.9936 | -0.0166 | 1 | 0 | 2 | 0 | 361.00 | 8.49 | 8.50 | 490000 | 14249114.30 | 35857348.10 | 1 | 36.82 | 3 | 2.80 |
| *Anas strepera* | 1.2302 | 0.4300 | 1 | 0 | 2 | 0 | 792.50 | 9.95 | 9.96 | 78000 | 17885864.40 | 62053837.00 | 2 | 17.28 | 1 | 4.55 |
| *Anser anser* | 2.2465 | 0.9593 | 0 | 1 | 2 | 1 | 3464.50 | 5.90 | 5.90 | 155000 | 22235990.80 | 51496171.00 | 2 | 12.28 | 9 | 11.08 |
| *Anthus campestris* | 0.9583 | 0.0573 | 0 | 0 | 0 | 0 | 28.80 | - | - | 1450000 | 15550721.80 | 55732972.00 | 3 | 16.62 | 0 | 0.52 |
| *Anthus pratensis* | 0.9470 | -0.0217 | 0 | 0 | 1 | 0 | 19.25 | 4.65 | 13.92 | 11500000 | 17595781.30 | 19961896.40 | 2 | 15.65 | 2 | 0.48 |
| *Anthus spinoletta* | 1.0102 | -0.0391 | 0 | 0 | 1 | 0 | 21.45 | 5.45 | 10.90 | 1520000 | 19601138.30 | 58944503.40 | 8 | 21.27 | 2 | 0.58 |
| *Anthus trivialis* | 0.9143 | -0.0087 | 0 | 0 | 0 | 0 | 23.40 | 4.53 | 13.59 | 34500000 | 18113964.60 | 43026565.70 | 3 | 47.07 | 3 | 0.62 |
| *Apus apus* | 0.9811 | 0.0045 | 0 | 1 | 0 | 1 | 39.65 | 2.40 | 2.40 | 11950000 | 18290318.80 | 44557537.40 | 2 | 59.39 | 1 | 0.61 |
| *Ardea cinerea* | 1.1216 | 0.0759 | 0 | 1 | 2 | 1 | 1433.00 | 3.95 | 7.90 | 250000 | 19718995.10 | 108370772.00 | 4 | 1.50 | 1 | 7.93 |
| *Ardea purpurea* | 1.1223 | 0.0531 | 0 | 1 | 2 | 0 | 873.75 | 4.50 | 4.50 | 35500 | 13298310.80 | 61038243.00 | 4 | - | 2 | - |
| *Asio otus* | 0.9495 | -0.0301 | 0 | 0 | 0 | 0 | 255.50 | 4.15 | 8.30 | 595000 | 20857811.60 | 57054530.00 | 6 | 3.18 | 5 | 5.68 |
| *Athene noctua* | 1.0778 | 0.0943 | 0 | 0 | 0 | 0 | 168.00 | 3.60 | 7.20 | 930000 | 14334507.70 | 73165615.80 | 12 | - | 5 | 3.34 |
| *Aythya ferina* | 0.9926 | -0.0368 | 1 | 0 | 2 | 0 | 869.00 | 9.51 | 9.50 | 325000 | 17531760.40 | 30655029.10 | 1 | 17.67 | - | 5.86 |
| *Aythya fuligula* | 0.9684 | 0.0161 | 1 | 0 | 2 | 0 | 656.50 | 10.89 | 10.90 | 805000 | 18581006.50 | 42202535.00 | 1 | 17.08 | 0 | 4.48 |
| *Branta leucopsis* | 1.3542 | 0.0190 | 0 | 1 | 2 | 0 | 1630.50 | 4.50 | 4.50 | 47500 | 2917574.20 | 3110671.50 | 1 | 20.25 | - | 6.75 |
| *Bubulcus ibis* | 1.1189 | 0.2297 | 0 | 1 | 1 | 0 | 307.50 | 4.50 | 9.00 | 60000 | 8949377.49 | 92711394.00 | 2 | - | - | 4.23 |
| *Bucephala clangula* | 0.8732 | 0.0017 | 1 | 0 | 2 | 0 | 840.30 | 9.51 | 9.50 | 540000 | 11242036.00 | 48836042.40 | 2 | 9.66 | 3 | 6.15 |
| *Burhinus oedicnemus* | 0.9929 | 0.1698 | 0 | 0 | 0 | 0 | 462.00 | 1.90 | 1.90 | 62000 | 13204270.30 | 62584206.10 | 6 | - | - | 3.50 |
| *Buteo buteo* | 0.9115 | -0.0014 | 0 | 0 | 0 | 0 | 806.50 | 2.53 | 2.53 | 955000 | 20992209.50 | 80859225.30 | 12 | 29.57 | 3 | 7.88 |
| *Calandrella brachydactyla* | 0.9393 | 0.0099 | 1 | 0 | 0 | 0 | 22.25 | 3.51 | 7.02 | 10650000 | 11534711.00 | 33632687.30 | 8 | 17.47 | 0 | - |
| *Carduelis carduelis* | 0.9807 | 0.0313 | 1 | 0 | 0 | 1 | 15.60 | 4.78 | 14.31 | 20500000 | 19353063.00 | 42127228.30 | 14 | 1.16 | 8 | 0.56 |
| *Carduelis citrinella* | 0.8719 | -0.0645 | 1 | 0 | 0 | 0 | 12.75 | 4.60 | 9.20 | 337000 | 1979731.22 | 1979731.22 | 2 | 1.09 | - | - |
| *Carpodacus erythrinus* | 0.9985 | 0.0097 | 1 | 0 | 0 | 0 | 23.40 | 5.13 | 5.13 | 4550000 | 13671547.70 | 56635635.00 | 5 | 24.22 | - | - |
| *Cecropis daurica* | 1.1200 | 0.0096 | 0 | 0 | 0 | 0 | 22.25 | 4.13 | 12.39 | 265000 | 13618405.80 | 123066917.00 | 11 | 5.00 | - | - |
| *Certhia brachydactyla* | 0.9083 | 0.0047 | 0 | 0 | 0 | 0 | 9.15 | 5.90 | 11.80 | 6200000 | 9568574.94 | 11981649.00 | 5 | 0.00 | - | 0.53 |
| *Certhia familiaris* | 0.9629 | -0.0043 | 0 | 0 | 0 | 0 | 9.15 | 5.43 | 10.86 | 8350000 | 18408794.00 | 52296727.30 | 22 | 0.00 | 3 | 0.50 |
| *Cettia cetti* | 0.9259 | -0.0097 | 0 | 0 | 1 | 0 | 14.10 | 4.55 | 9.10 | 1100000 | 10980957.00 | 22945867.40 | 3 | 2.11 | 0 | - |
| *Charadrius dubius* | 1.3168 | 0.0013 | 1 | 0 | 1 | 0 | 38.75 | 3.87 | 7.74 | 175000 | 19241851.00 | 56955930.00 | 3 | 34.02 | - | 0.78 |
| *Chloris chloris* | 0.9081 | 0.0256 | 1 | 0 | 0 | 1 | 27.65 | 4.83 | 9.66 | 23000000 | 22122014.70 | 33683562.90 | 10 | 1.34 | 8 | 0.88 |
| *Chroicocephalus ridibundus* | 1.1256 | 0.0565 | 0 | 1 | 2 | 0 | 280.50 | 2.54 | 2.54 | 1850000 | 19694179.40 | 43657220.40 | 1 | 23.50 | 1 | 2.76 |
| *Ciconia ciconia* | 1.0158 | 0.0314 | 0 | 1 | 1 | 1 | 3448.00 | 4.00 | 4.00 | 200000 | 14362291.30 | 38978214.10 | 3 | 41.87 | 0 | 14.92 |
| *Cinclus cinclus* | 1.2075 | -0.0395 | 0 | 0 | 2 | 0 | 61.90 | 4.42 | 8.84 | 250000 | 18329712.50 | 32649233.40 | 14 | 0.00 | 2 | 1.44 |
| *Circaetus gallicus* | 1.0948 | -0.0552 | 0 | 0 | 0 | 0 | 1699.50 | 1.00 | 1.00 | 10700 | 14757483.20 | 51628656.40 | 1 | - | 0 | - |
| *Circus aeruginosus* | 1.0843 | -0.0097 | 1 | 1 | 1 | 0 | 584.50 | 4.66 | 4.66 | 116500 | 16683323.40 | 55289894.00 | 9 | 6.42 | 3 | 5.62 |
| *Circus cyaneus* | 0.9707 | -0.0276 | 1 | 0 | 0 | 0 | 436.50 | 4.46 | 4.46 | 45500 | 16284555.00 | 80205966.00 | 4 | 52.55 | 1 | 4.60 |
| *Circus pygargus* | 1.0460 | -0.0176 | 1 | 0 | 0 | 0 | 315.50 | 4.20 | 4.20 | 50000 | 14687885.80 | 24877885.40 | 1 | - | 0 | - |
| *Cisticola juncidis* | 1.0274 | 0.0045 | 0 | 0 | 0 | 0 | 8.50 | 5.70 | 17.10 | 665000 | 11176227.50 | 90176000.00 | 17 | 0.00 | 0 | - |
| *Clamator glandarius* | 1.0930 | 0.1367 | 0 | 0 | 0 | 0 | 153.50 | 6.00 | 17.99 | 67500 | 6288955.78 | 59374966.60 | 2 | 4.58 | 0 | 1.80 |
| *Coccothraustes coccothraustes* | 0.9753 | 0.0058 | 1 | 0 | 0 | 1 | 54.70 | 4.60 | 4.60 | 3300000 | 15350032.80 | 46328894.70 | 5 | 5.07 | 6 | 1.69 |
| *Coloeus monedula* | 1.0121 | 0.0236 | 0 | 0 | 0 | 1 | 249.00 | 4.40 | 4.40 | 10100000 | 17130066.10 | 31652899.30 | 4 | 0.29 | 3 | 4.40 |
| *Columba livia* | 0.9991 | -0.0049 | 0 | 1 | 0 | 1 | 261.00 | 1.93 | 9.65 | 12150000 | 22011568.70 | 102111594.00 | 9 | - | 3 | 2.23 |
| *Columba oenas* | 1.0697 | 0.1669 | 0 | 0 | 0 | 0 | 314.50 | 2.30 | 9.20 | 625000 | 16474024.50 | 31125951.50 | 2 | 3.45 | 1 | 2.27 |
| *Columba palumbus* | 0.8449 | 0.0067 | 0 | 0 | 0 | 1 | 494.50 | 1.84 | 5.52 | 13000000 | 20992209.50 | 42222968.80 | 5 | 2.04 | 4 | 2.19 |
| *Coracias garrulus* | 1.0024 | 0.0168 | 0 | 0 | 0 | 0 | 147.00 | 3.80 | 3.80 | 81500 | 14693843.50 | 27947574.30 | 2 | 55.75 | 2 | 2.20 |
| *Corvus corax* | 1.1245 | 0.0479 | 0 | 0 | 0 | 0 | 1200.55 | 4.50 | 4.50 | 710000 | 24716372.70 | 116351764.00 | 9 | 0.00 | 3 | 15.37 |
| *Corvus corone* | 0.8678 | 0.0089 | 0 | 0 | 0 | 1 | 544.50 | 4.10 | 4.10 | 12000000 | 18650577.80 | 57070552.40 | 6 | 5.72 | 4 | 8.12 |
| *Corvus frugilegus* | 1.1478 | 0.0974 | 0 | 1 | 0 | 1 | 453.50 | 4.45 | 4.45 | 14000000 | 16762116.70 | 47448563.00 | 2 | 2.32 | 4 | 7.98 |
| *Coturnix coturnix* | 1.1352 | 0.0500 | 1 | 0 | 0 | 0 | 98.75 | 10.21 | 20.40 | 3750000 | 18225583.10 | 57146986.00 | 5 | 9.25 | 2 | - |
| *Crex crex* | 1.1279 | -0.0523 | 0 | 0 | 1 | 0 | 146.50 | 8.89 | 17.80 | 1650000 | 13672372.80 | 24591321.90 | 1 | 67.92 | - | 1.15 |
| *Cuculus canorus* | 0.9255 | -0.0056 | 0 | 0 | 0 | 0 | 120.50 | 9.20 | 9.20 | 6400000 | 18418484.90 | 79987915.80 | 4 | 49.38 | 0 | 1.44 |
| *Cyanistes caeruleus* | 0.9115 | 0.0448 | 1 | 0 | 0 | 1 | 11.75 | 11.75 | 23.52 | 32000000 | 18836186.10 | 22374181.10 | 15 | 0.00 | 6 | 0.60 |
| *Cyanopica cyanus* | 0.9441 | 0.0662 | 0 | 0 | 0 | 0 | 71.00 | 6.58 | 6.58 | 360000 | 1194274.23 | 1669659.27 | 11 | 0.00 | 3 | 2.06 |
| *Cygnus cygnus* | 1.0461 | 0.0147 | 0 | 0 | 2 | 0 | 11375.00 | 4.40 | 4.40 | 18500 | 13456830.50 | 32594299.70 | 1 | 9.29 | 0 | - |
| *Cygnus olor* | 1.0266 | 0.0465 | 1 | 1 | 2 | 0 | 10750.00 | 6.00 | 6.00 | 103000 | 14960076.20 | 30854759.80 | 1 | 1.08 | 1 | 16.03 |
| *Delichon urbicum* | 0.9734 | -0.0126 | 0 | 1 | 0 | 1 | 19.55 | 4.10 | 8.20 | 16950000 | 18408794.00 | 86520645.40 | 5 | 44.25 | 6 | 0.44 |
| *Dendrocopos major* | 0.9397 | 0.0186 | 1 | 0 | 0 | 1 | 89.65 | 5.50 | 5.50 | 15000000 | 19513931.70 | 79065128.10 | 26 | 0.00 | 0 | 2.58 |
| *Dendrocopos medius* | 1.0415 | 0.0224 | 1 | 0 | 0 | 0 | 59.00 | 5.64 | 5.64 | 225000 | 11961770.00 | 14754777.50 | 4 | - | - | 1.79 |
| *Dendrocopos minor* | 1.0525 | 0.0241 | 1 | 0 | 0 | 0 | 25.50 | 5.00 | 5.00 | 775000 | 18229498.50 | 45996197.60 | 14 | 0.00 | - | 1.20 |
| *Dendrocopos syriacus* | 1.0085 | 0.0393 | 1 | 0 | 0 | 0 | 76.75 | 5.11 | 5.10 | 815000 | 6429844.05 | 9427111.87 | 3 | - | - | - |
| *Dryocopus martius* | 0.9531 | 0.0234 | 1 | 0 | 0 | 0 | 273.00 | 4.80 | 4.80 | 1070000 | 17126959.60 | 54009694.80 | 2 | 0.00 | - | 7.12 |
| *Egretta garzetta* | 1.0289 | 0.0427 | 0 | 1 | 2 | 0 | 532.50 | 4.80 | 4.80 | 81000 | 10944631.70 | 142245902.00 | 4 | 7.53 | 1 | 3.70 |
| *Emberiza calandra* | 0.8758 | 0.0135 | 0 | 0 | 0 | 0 | 47.65 | 3.97 | 11.91 | 14950000 | 15792159.10 | 26299101.40 | 3 | 6.41 | 2 | 1.18 |
| *Emberiza cia* | 0.9156 | 0.0322 | 1 | 0 | 0 | 0 | 23.25 | 3.78 | 11.36 | 2700000 | 10609419.00 | 21375694.70 | 4 | 0.00 | 1 | - |
| *Emberiza cirlus* | 0.8494 | 0.0237 | 1 | 0 | 0 | 0 | 23.75 | 3.53 | 10.59 | 3600000 | 7281464.52 | 10526563.20 | 1 | 0.48 | 3 | 0.79 |
| *Emberiza citrinella* | 0.7533 | -0.0153 | 1 | 0 | 0 | 0 | 26.75 | 4.49 | 13.47 | 24500000 | 15714281.40 | 26826062.90 | 3 | 4.72 | 4 | 0.77 |
| *Emberiza hortulana* | 0.8773 | -0.0045 | 1 | 0 | 0 | 0 | 20.60 | 4.52 | 4.52 | 10600000 | 16908047.10 | 27681228.40 | 1 | 36.48 | 0 | - |
| *Emberiza rustica* | 1.1143 | -0.0005 | 1 | 0 | 0 | 0 | 19.05 | 4.50 | 9.00 | 8050000 | 5463913.21 | 19690540.10 | 2 | 27.06 | - | - |
| *Emberiza schoeniclus* | 0.9444 | 0.0376 | 1 | 0 | 1 | 0 | 18.80 | 4.76 | 9.52 | 6800000 | 18653395.00 | 55690607.50 | 18 | 10.52 | 3 | 0.75 |
| *Erithacus rubecula* | 0.8835 | -0.0015 | 0 | 0 | 0 | 1 | 16.35 | 5.00 | 15.00 | 63000000 | 22011568.70 | 38251134.10 | 8 | 5.00 | 5 | 0.62 |
| *Falco naumanni* | 1.1356 | 0.1572 | 1 | 1 | 0 | 1 | 152.00 | 3.83 | 3.83 | 33500 | 13134238.50 | 30494410.50 | 1 | - | 1 | 2.90 |
| *Falco peregrinus* | 1.2293 | 0.3676 | 1 | 0 | 0 | 1 | 889.25 | 3.40 | 3.40 | 18500 | 23278713.00 | 50141360.00 | 15 | 15.11 | 3 | 6.79 |
| *Falco subbuteo* | 0.9839 | -0.0025 | 1 | 0 | 0 | 0 | 211.00 | 2.93 | 2.93 | 95500 | 16906896.10 | 66368744.50 | 2 | 41.74 | 2 | 3.50 |
| *Falco tinnunculus* | 1.0417 | -0.0302 | 1 | 1 | 0 | 1 | 174.50 | 4.72 | 4.72 | 415000 | 22234285.70 | 115701982.00 | 11 | 5.60 | 8 | 3.72 |
| *Ficedula albicollis* | 1.0182 | 0.1301 | 1 | 0 | 0 | 0 | 13.40 | 5.79 | 5.80 | 1900000 | 7661786.28 | 7661786.28 | 1 | 61.28 | - | - |
| *Ficedula hypoleuca* | 0.9648 | -0.0138 | 1 | 0 | 0 | 1 | 14.35 | 7.45 | 7.45 | 16000000 | 17937280.20 | 31222685.20 | 4 | 43.00 | 5 | 0.38 |
| *Fringilla coelebs* | 0.6931 | 0.0155 | 1 | 0 | 0 | 1 | 24.20 | 4.30 | 8.60 | 185000000 | 22446773.90 | 43879941.30 | 17 | 5.54 | 7 | 0.81 |
| *Fringilla montifringilla* | 1.0173 | 0.0031 | 1 | 0 | 0 | 0 | 22.65 | 5.79 | 11.60 | 17500000 | 7076887.60 | 21206352.40 | 1 | 13.75 | 4 | 0.70 |
| *Fulica atra* | 1.1208 | 0.0441 | 0 | 0 | 2 | 0 | 732.50 | 7.46 | 14.92 | 1800000 | 20439355.60 | 69071577.00 | 4 | 3.32 | 0 | 3.08 |
| *Galerida cristata* | 0.9077 | -0.0247 | 0 | 0 | 0 | 1 | 44.65 | 3.90 | 11.70 | 5600000 | 14667778.70 | 83319431.40 | 48 | 0.00 | 0 | 1.10 |
| *Galerida theklae* | 0.9154 | 0.0237 | 0 | 0 | 0 | 0 | 35.60 | 4.60 | 13.80 | 1800000 | 5077272.97 | 25706180.90 | 12 | 0.00 | - | 0.92 |
| *Gallinago gallinago* | 1.0346 | -0.0333 | 0 | 0 | 1 | 0 | 106.50 | 3.89 | 3.89 | 1415000 | 22446773.90 | 100959752.00 | 9 | 7.05 | 1 | 1.34 |
| *Gallinula chloropus* | 1.0882 | -0.0256 | 0 | 0 | 2 | 1 | 348.50 | 6.58 | 19.73 | 1300000 | 20127917.93 | 107603464.00 | 12 | 2.13 | 0 | 1.90 |
| *Garrulus glandarius* | 0.9782 | 0.0253 | 0 | 0 | 0 | 1 | 161.70 | 4.80 | 4.80 | 9500000 | 17963092.00 | 70688189.00 | 43 | 0.00 | 4 | 3.97 |
| *Gavia arctica* | 0.9578 | 0.0135 | 0 | 0 | 2 | 0 | 2804.50 | 1.88 | 1.88 | 71500 | 13865633.70 | 52965781.40 | 3 | 10.57 | - | 7.50 |
| *Gavia stellata* | 1.0206 | -0.0101 | 0 | 1 | 2 | 0 | 1603.00 | 1.79 | 1.79 | 62000 | 14074290.40 | 66565236.60 | 1 | 17.29 | - | 5.39 |
| *Grus grus* | 1.0459 | 0.0409 | 0 | 0 | 1 | 0 | 4541.50 | 2.00 | 2.00 | 92000 | 12797426.90 | 34386572.20 | 2 | 39.10 | - | 17.60 |
| *Gyps fulvus* | 0.8250 | 0.0304 | 0 | 1 | 0 | 0 | 9250.00 | 1.00 | 1.00 | 20000 | 6846263.27 | 32363011.00 | 2 | - | 0 | 26.00 |
| *Haematopus ostralegus* | 0.8884 | -0.0039 | 0 | 0 | 1 | 1 | 531.00 | 2.78 | 2.78 | 375000 | 20998321.20 | 46951330.50 | 3 | 21.39 | 0 | 3.78 |
| *Himantopus himantopus* | 1.3176 | 0.1403 | 0 | 1 | 2 | 0 | 178.75 | 4.00 | 4.00 | 50500 | 12445086.80 | 72025176.00 | 5 | - | 0 | - |
| *Hippolais icterina* | 0.9115 | -0.0041 | 0 | 0 | 0 | 1 | 13.30 | 5.04 | 5.04 | 5300000 | 14133264.80 | 21131335.80 | 1 | 71.34 | 3 | 0.48 |
| *Hippolais polyglotta* | 0.9103 | 0.0120 | 0 | 0 | 0 | 0 | 11.45 | 4.33 | 8.68 | 2000000 | 3902696.90 | 5102646.74 | 1 | 30.63 | 2 | - |
| *Hirundo rustica* | 0.8835 | -0.0159 | 1 | 1 | 0 | 1 | 19.10 | 4.40 | 13.23 | 26000000 | 18423469.70 | 79406476.50 | 6 | 42.34 | 4 | 0.55 |
| *Ichthyaetus melanocephalus* | 1.1716 | 0.1460 | 0 | 1 | 2 | 0 | - | 3.00 | 3.00 | 220000 | 5495226.13 | 5495226.13 | 1 | - | - | - |
| *Jynx torquilla* | 1.0220 | 0.0091 | 0 | 0 | 0 | 0 | 37.35 | 8.20 | 24.59 | 940000 | 17856896.80 | 44408201.00 | 8 | 35.20 | 3 | 0.85 |
| *Lagopus lagopus* | 1.1279 | -0.0324 | 1 | 0 | 0 | 0 | 550.50 | 7.50 | 7.50 | 2700000 | 10682560.20 | 30623543.10 | 16 | - | 4 | 2.51 |
| *Lanius collurio* | 0.9114 | 0.0140 | 1 | 0 | 0 | 0 | 30.70 | 4.44 | 4.44 | 9650000 | 9350266.22 | 10296961.40 | 3 | 64.73 | 5 | 1.04 |
| *Lanius excubitor* | 1.0143 | 0.0124 | 0 | 0 | 0 | 0 | 68.30 | 6.19 | 6.20 | - | - | - | - | - | 4 | 1.78 |
| *Lanius meridionalis* | 0.9134 | -0.0438 | 0 | 0 | 0 | 0 | 51.65 | 5.04 | - | - | - | - | - | - | - | - |
| *Lanius minor* | 1.0360 | -0.0669 | 1 | 0 | 0 | 0 | 46.55 | 6.17 | 6.17 | 1060000 | 10527588.60 | 22100450.90 | 1 | 62.59 | 1 | - |
| *Lanius senator* | 0.8355 | 0.0073 | 1 | 0 | 0 | 0 | 36.00 | 5.38 | 5.38 | 840000 | 11686313.70 | 16564816.00 | 4 | 28.45 | 2 | - |
| *Larus argentatus* | 0.9590 | 0.0460 | 0 | 1 | 2 | 1 | 895.00 | 3.00 | 3.00 | - | - | - | - | - | 2 | 5.59 |
| *Larus canus* | 0.9242 | 0.0188 | 0 | 1 | 2 | 0 | 386.50 | 3.00 | 3.00 | 1045000 | 12299926.40 | 41869021.50 | 4 | 13.76 | 0 | 3.95 |
| *Larus fuscus* | 1.0650 | 0.1077 | 0 | 1 | 2 | 0 | 817.50 | 2.71 | 2.71 | 325000 | 18673624.70 | 30475762.70 | 5 | 34.35 | 0 | 5.70 |
| *Larus marinus* | 1.1267 | 0.2867 | 0 | 1 | 2 | 0 | 1599.50 | 2.90 | 2.90 | 145000 | 24007036.50 | 39787925.80 | 1 | 7.28 | - | 7.46 |
| *Larus michahellis* | 1.0151 | -0.0467 | 0 | 1 | 2 | 1 | 1154.00 | - | - | - | - | - | - | - | - | - |
| *Limosa limosa* | 0.9065 | -0.0641 | 0 | 0 | 1 | 0 | 331.50 | 3.86 | 3.86 | 119500 | 15130744.50 | 35726623.60 | 3 | 50.60 | - | 2.90 |
| *Linaria cannabina* | 0.9366 | -0.0255 | 1 | 0 | 0 | 1 | 18.95 | 4.40 | 13.23 | 19000000 | 18741686.80 | 35387964.50 | 7 | 4.11 | 6 | 0.65 |
| *Locustella fluviatilis* | 0.9239 | -0.0026 | 0 | 0 | 1 | 0 | 18.80 | 5.00 | 5.00 | 3250000 | 7968923.67 | 9966147.64 | 1 | 63.42 | - | - |
| *Locustella luscinioides* | 0.9603 | 0.0085 | 0 | 0 | 1 | 0 | 16.60 | 5.00 | 10.00 | 665000 | 13776444.80 | 30803045.10 | 3 | 29.94 | 0 | - |
| *Locustella naevia* | 0.9596 | -0.0109 | 0 | 0 | 0 | 0 | 12.70 | 5.45 | 16.38 | 1520000 | 15493382.40 | 24571176.70 | 4 | 29.75 | 0 | 0.60 |
| *Lophophanes cristatus* | 0.9638 | -0.0043 | 0 | 0 | 0 | 0 | 11.15 | 6.50 | 13.00 | 9050000 | 16272354.80 | 17444545.80 | 7 | 0.00 | 0 | 0.66 |
| *Loxia curvirostra* | 1.1956 | -0.0115 | 1 | 0 | 0 | 0 | 40.60 | 3.70 | 11.10 | 9400000 | 17613905.30 | 69460519.50 | 18 | 0.00 | 5 | 1.55 |
| *Lullula arborea* | 0.9913 | 0.0089 | 0 | 0 | 0 | 0 | 30.05 | 3.97 | 7.94 | 2300000 | 14535016.60 | 17661276.50 | 2 | 3.80 | 2 | - |
| *Luscinia luscinia* | 0.9392 | 0.0147 | 0 | 0 | 0 | 0 | 25.00 | 4.80 | 4.80 | 5300000 | 10193761.00 | 16872174.90 | 1 | 63.10 | 1 | 0.75 |
| *Luscinia megarhynchos* | 0.8919 | 0.0223 | 0 | 0 | 0 | 0 | 20.15 | 4.75 | 9.50 | 8100000 | 11843585.00 | 24692307.50 | 3 | 32.20 | 1 | - |
| *Luscinia svecica* | 1.0094 | 0.0043 | 1 | 0 | 0 | 0 | 18.25 | 6.19 | 12.38 | 6150000 | 17624545.50 | 63131558.80 | 10 | 25.50 | 2 | - |
| *Lyrurus tetrix* | 1.0399 | 0.0155 | 1 | 0 | 0 | 0 | 1107.00 | 7.91 | 7.91 | 2850000 | 13396794.40 | 29140443.00 | 7 | - | 5 | 3.52 |
| *Melanocorypha calandra* | 0.8780 | -0.0211 | 0 | 0 | 0 | 0 | 60.40 | 4.20 | 8.40 | 17000000 | 10563438.80 | 23008892.20 | 3 | 0.00 | 2 | 1.36 |
| *Mergus merganser* | 0.9375 | 0.0135 | 1 | 0 | 2 | 0 | 1641.50 | 9.51 | 9.50 | 60500 | 22283044.50 | 92714908.00 | 3 | 4.89 | 2 | 7.01 |
| *Mergus serrator* | 0.9468 | 0.0081 | 1 | 0 | 2 | 0 | 1090.50 | 9.20 | 9.20 | 96500 | 18052044.90 | 77015014.50 | 1 | 11.06 | 2 | 4.92 |
| *Merops apiaster* | 1.1945 | -0.0135 | 0 | 1 | 0 | 0 | 55.10 | 6.00 | 6.00 | 740000 | 13583929.50 | 94226129.10 | 1 | 19.86 | 1 | 0.80 |
| *Milvus migrans* | 1.1566 | -0.0095 | 0 | 1 | 0 | 0 | 828.50 | 2.50 | 2.50 | 82000 | 20337192.20 | 131473244.00 | 6 | 15.79 | 0 | 6.60 |
| *Milvus milvus* | 1.0613 | 0.2487 | 0 | 0 | 0 | 0 | 1015.50 | 2.00 | 2.00 | 22000 | 13971044.10 | 27521008.80 | 2 | - | 3 | 7.30 |
| *Monticola saxatilis* | 1.1867 | 0.0429 | 1 | 0 | 0 | 0 | 51.25 | 4.50 | 9.00 | 210000 | 13405910.10 | 29812419.20 | 1 | 38.38 | 3 | - |
| *Monticola solitarius* | 1.1816 | 0.3899 | 1 | 0 | 0 | 0 | 51.25 | 4.54 | 9.08 | 190000 | 9208282.29 | 86047026.50 | 5 | 9.31 | - | 1.36 |
| *Motacilla alba* | 0.8683 | -0.0048 | 1 | 0 | 0 | 1 | 20.75 | 5.11 | 15.30 | 19500000 | 22419730.10 | 96194964.20 | 11 | 18.13 | 4 | 0.53 |
| *Motacilla cinerea* | 0.9848 | -0.0148 | 1 | 0 | 1 | 0 | 17.35 | 4.90 | 14.70 | 1170000 | 22129083.40 | 62906101.60 | 6 | 24.68 | 1 | 0.53 |
| *Motacilla flava* | 0.9348 | -0.0051 | 1 | 0 | 1 | 0 | 17.45 | 5.20 | 10.40 | 10950000 | 20154247.50 | 95983445.10 | 18 | 40.99 | 4 | 0.43 |
| *Muscicapa striata* | 0.9564 | -0.0141 | 0 | 0 | 0 | 1 | 15.50 | 4.25 | 8.50 | 18000000 | 18450713.60 | 39661505.30 | 7 | 64.40 | 3 | 0.48 |
| *Nucifraga caryocatactes* | 1.2360 | -0.0445 | 0 | 0 | 0 | 0 | 193.00 | 3.50 | 3.50 | 630000 | 13736314.00 | 60803988.50 | 9 | 0.00 | 0 | 5.50 |
| *Numenius arquata* | 0.8658 | 0.0155 | 0 | 0 | 1 | 0 | 725.00 | 3.78 | 3.78 | 290000 | 13184494.20 | 25798929.20 | 2 | 44.67 | 0 | 4.05 |
| *Numenius phaeopus* | 1.0203 | 0.0148 | 0 | 0 | 1 | 0 | 383.00 | 3.88 | 3.88 | 260000 | 10749418.16 | 30525640.40 | 4 | 70.56 | 0 | 1.70 |
| *Nycticorax nycticorax* | 1.2003 | 0.1429 | 0 | 1 | 2 | 0 | 636.00 | 4.00 | 4.00 | 75000 | 11631510.60 | 133654200.00 | 4 | 9.09 | 3 | 5.90 |
| *Oenanthe hispanica* | 0.9448 | -0.0199 | 1 | 0 | 0 | 0 | 15.10 | 5.28 | 10.58 | 2350000 | 7457029.20 | 12349013.70 | 2 | 16.57 | 1 | - |
| *Oenanthe leucura* | 1.0592 | -0.0106 | 1 | 0 | 0 | 0 | - | 4.00 | 8.00 | 10050 | 2321398.00 | 6367277.36 | 2 | 0.00 | 0 | - |
| *Oenanthe oenanthe* | 0.9767 | 0.0071 | 1 | 0 | 0 | 0 | 23.95 | 5.50 | 11.00 | 8800000 | 22419730.10 | 84869865.30 | 4 | 38.18 | 3 | 0.68 |
| *Oriolus oriolus* | 0.8341 | 0.0080 | 1 | 0 | 0 | 0 | 68.50 | 3.80 | 3.80 | 5250000 | 15491271.10 | 37953231.90 | 2 | 44.77 | 3 | 1.31 |
| *Pandion haliaetus* | 0.9750 | -0.0270 | 0 | 1 | 2 | 0 | 1527.50 | 2.63 | 2.63 | 9300 | 22011568.70 | 75521650.00 | 5 | 13.42 | - | 9.60 |
| *Parus major* | 0.8421 | -0.0076 | 1 | 0 | 0 | 1 | 18.50 | 10.91 | 21.84 | 68500000 | 18706806.10 | 124262164.00 | 31 | 0.00 | 5 | 0.85 |
| *Passer domesticus* | 0.8951 | -0.0040 | 1 | 1 | 0 | 1 | 30.35 | 4.00 | 16.00 | 96500000 | 19222360.90 | 92054601.80 | 12 | 0.00 | 6 | 0.90 |
| *Passer montanus* | 0.9906 | -0.0109 | 0 | 1 | 0 | 1 | 21.70 | 4.92 | 14.76 | 37000000 | 18340824.10 | 127746779.00 | 10 | 3.62 | 5 | 0.76 |
| *Perdix perdix* | 1.0072 | 0.0041 | 1 | 0 | 0 | 0 | 382.00 | 14.59 | 14.60 | 2350000 | 15345200.00 | 22588134.50 | 8 | 0.00 | 4 | 1.55 |
| *Periparus ater* | 0.9289 | 0.0090 | 0 | 0 | 0 | 0 | 9.25 | 8.49 | 17.00 | 20500000 | 17463048.60 | 67081982.90 | 20 | 0.00 | 6 | 0.57 |
| *Perisoreus infaustus* | 1.0105 | 0.0170 | 0 | 0 | 0 | 0 | 84.70 | 3.70 | 3.70 | 525000 | 9354487.55 | 27954937.80 | 16 | 0.00 | 1 | - |
| *Pernis apivorus* | 1.0697 | 0.0032 | 0 | 0 | 0 | 0 | 626.00 | 2.00 | 2.00 | 135000 | 16481731.40 | 24169813.40 | 1 | 60.75 | 1 | 7.64 |
| *Petronia petronia* | 0.8387 | -0.0025 | 1 | 1 | 0 | 0 | 32.90 | 5.70 | 5.70 | 2750000 | 12273403.90 | 32839061.90 | 7 | 0.00 | 3 | - |
| *Phalacrocorax carbo* | 1.2759 | 0.6346 | 0 | 1 | 2 | 0 | 2254.00 | 3.50 | 3.50 | 340000 | 22453517.60 | 109012925.00 | 5 | 2.64 | - | 9.86 |
| *Philomachus pugnax* | 0.9538 | 0.0079 | 1 | 0 | 1 | 0 | 140.50 | 3.72 | 3.72 | 355000 | 7637306.47 | 21967652.00 | 1 | 52.21 | - | 1.70 |
| *Phoenicurus ochruros* | 0.8653 | 0.0071 | 1 | 0 | 0 | 1 | 16.00 | 4.90 | 14.70 | 6400000 | 14506612.20 | 35108159.90 | 7 | 15.83 | 1 | 0.55 |
| *Phoenicurus phoenicurus* | 0.9968 | 0.0201 | 1 | 0 | 0 | 1 | 15.90 | 6.04 | 12.08 | 11400000 | 18043482.20 | 39333277.90 | 2 | 33.93 | 4 | 0.49 |
| *Phylloscopus bonelli* | 0.9480 | 0.0411 | 0 | 0 | 0 | 0 | 7.75 | 5.30 | 10.60 | 2450000 | 8596743.76 | 11145974.80 | 2 | 26.86 | 3 | - |
| *Phylloscopus collybita* | 0.9219 | 0.0092 | 0 | 0 | 0 | 1 | 7.70 | 5.50 | 11.00 | 45445000 | 20356151.00 | 57078465.00 | 6 | 22.55 | 4 | 0.36 |
| *Phylloscopus sibilatrix* | 0.9488 | -0.0236 | 0 | 0 | 0 | 0 | 9.10 | 5.90 | 11.80 | 18000000 | 14172995.80 | 20758794.10 | 1 | 52.75 | 1 | - |
| *Phylloscopus trochiloides* | 1.1268 | 0.0981 | 0 | 0 | 0 | 0 | 7.05 | 4.70 | 4.70 | 13500000 | 11333570.90 | 33405236.40 | 4 | 26.79 | - | - |
| *Phylloscopus trochilus* | 0.8225 | -0.0132 | 0 | 0 | 0 | 0 | 9.35 | 6.19 | 12.40 | 78000000 | 13133242.10 | 34702774.70 | 3 | 68.09 | 8 | 0.31 |
| *Pica pica* | 0.8994 | -0.0081 | 0 | 0 | 0 | 1 | 228.00 | 5.90 | 5.90 | 13250000 | 19417779.90 | 125873841.00 | 12 | 0.00 | 5 | 5.58 |
| *Picus canus* | 1.0827 | -0.0472 | 1 | 0 | 0 | 0 | 136.50 | 8.00 | 8.00 | 250000 | 14645722.60 | 102686392.00 | 13 | 0.00 | - | 3.60 |
| *Picus viridis* | 0.9480 | 0.1076 | 1 | 0 | 0 | 1 | 193.50 | 6.10 | 6.10 | 945000 | 15594026.00 | 15764791.70 | 4 | 0.00 | 2 | 4.32 |
| *Pluvialis apricaria* | 1.0446 | 0.0096 | 0 | 0 | 1 | 0 | 175.50 | 3.85 | 3.85 | 600000 | 10337707.10 | 15646226.30 | 1 | 17.97 | 0 | 2.20 |
| *Podiceps auritus* | 1.2599 | 0.0449 | 0 | 1 | 2 | 0 | 394.00 | 4.50 | 4.50 | 8650 | 14349206.50 | 47747524.00 | 1 | 10.44 | - | 2.40 |
| *Podiceps cristatus* | 1.0286 | -0.0161 | 0 | 1 | 2 | 0 | 875.00 | 3.50 | 7.00 | 375000 | 16832410.50 | 162810786.00 | 3 | 4.41 | - | 3.76 |
| *Podiceps grisegena* | 0.9632 | 0.0152 | 0 | 0 | 2 | 0 | 829.75 | 4.50 | 9.00 | 44000 | 11821761.80 | 50645660.70 | 2 | 7.87 | - | 2.94 |
| *Poecile montanus* | 0.9501 | -0.0185 | 0 | 0 | 0 | 0 | 11.65 | 8.05 | 8.05 | 33000000 | 17013325.80 | 57808042.00 | 11 | 0.00 | 2 | 0.79 |
| *Poecile palustris* | 0.9450 | 0.0072 | 0 | 0 | 0 | 0 | 11.90 | 8.02 | 16.04 | 4500000 | 15958622.90 | 60477602.70 | 16 | 0.00 | 1 | 0.72 |
| *Prunella collaris* | 1.0237 | -0.0856 | 0 | 0 | 0 | 0 | 42.25 | 3.50 | 7.00 | 140000 | 12973326.10 | 43136323.90 | 11 | 0.00 | 1 | 0.97 |
| *Prunella modularis* | 0.8707 | -0.0013 | 0 | 0 | 0 | 1 | 18.95 | 5.11 | 15.30 | 19000000 | 17187814.00 | 18168514.70 | 7 | 9.23 | 5 | 0.68 |
| *Ptyonoprogne rupestris* | 1.1218 | 0.0466 | 0 | 1 | 0 | 0 | 20.55 | 3.20 | 6.40 | 245000 | 9448795.78 | 50741090.00 | 1 | 0.95 | 0 | - |
| *Pyrrhocorax graculus* | 1.0143 | -0.0576 | 0 | 1 | 0 | 0 | 230.35 | 4.20 | 4.20 | 220000 | 10388483.40 | 25554494.30 | 3 | 0.00 | 0 | 3.20 |
| *Pyrrhocorax pyrrhocorax* | 1.0558 | -0.0206 | 0 | 0 | 0 | 0 | 330.00 | 4.70 | 4.70 | 76500 | 14141783.50 | 71209933.70 | 8 | 0.00 | - | 5.90 |
| *Pyrrhula pyrrhula* | 1.0133 | -0.0162 | 1 | 0 | 0 | 0 | 31.05 | 4.67 | 14.01 | 10650000 | 18402913.70 | 48033840.60 | 10 | 0.00 | 4 | 0.87 |
| *Rallus aquaticus* | 1.0550 | 0.0405 | 0 | 0 | 1 | 0 | 115.50 | 8.49 | 17.00 | 250000 | 20284841.80 | 61237984.50 | 4 | 2.69 | 1 | 1.76 |
| *Recurvirostra avosetta* | 1.2305 | -0.0503 | 0 | 1 | 2 | 0 | 282.00 | 3.90 | 3.90 | 47500 | 14579449.30 | 136890066.00 | 1 | 3.03 | - | 2.00 |
| *Regulus ignicapilla* | 0.9351 | 0.0591 | 1 | 0 | 0 | 0 | 5.25 | 8.79 | 17.64 | 5000000 | 9433511.61 | 11619888.70 | 3 | 1.61 | 1 | - |
| *Regulus regulus* | 0.9616 | -0.0158 | 1 | 0 | 0 | 0 | 5.80 | 10.35 | 20.72 | 27000000 | 19971090.90 | 60111317.80 | 13 | 0.00 | 3 | 0.33 |
| *Remiz pendulinus* | 1.1616 | -0.1397 | 1 | 0 | 1 | 0 | 9.15 | 4.40 | 8.80 | 315000 | 13988145.60 | 34607944.00 | 11 | 10.32 | - | 0.45 |
| *Riparia riparia* | 1.1033 | -0.0175 | 0 | 1 | 0 | 0 | 13.15 | 4.78 | 9.56 | 7450000 | 18437661.30 | 71056288.20 | 4 | 42.73 | 3 | 0.34 |
| *Saxicola rubetra* | 0.9387 | -0.0036 | 1 | 0 | 0 | 0 | 16.60 | 5.66 | 11.32 | 7700000 | 16611916.10 | 23515353.80 | 1 | 34.84 | 3 | 0.67 |
| *Saxicola torquatus* | 1.0334 | -0.0022 | 1 | 0 | 0 | 0 | 14.90 | 5.06 | 20.24 | 3300000 | 17345529.90 | 92401797.00 | 24 | 3.98 | - | - |
| *Scolopax rusticola* | 1.0355 | 0.0150 | 0 | 0 | 0 | 0 | 309.50 | 3.84 | 3.84 | 4200000 | 22068779.80 | 61514141.70 | 1 | 14.32 | 3 | 2.45 |
| *Serinus serinus* | 0.8732 | -0.0370 | 1 | 0 | 0 | 1 | 11.95 | 4.00 | 8.00 | 14150000 | 12823701.50 | 17598254.50 | 1 | 4.91 | 4 | 0.44 |
| *Sitta europaea* | 0.9800 | 0.0185 | 1 | 0 | 0 | 0 | 23.90 | 7.10 | 7.10 | 13250000 | 16995435.70 | 95378950.20 | 22 | 0.00 | 3 | 0.98 |
| *Somateria mollissima* | 0.9861 | 0.0046 | 1 | 1 | 2 | 0 | 2066.50 | 4.60 | 4.60 | 1020000 | 18285353.94 | 30201352.40 | 6 | 3.35 | 3 | 7.17 |
| *Spinus spinus* | 1.1223 | 0.1630 | 1 | 0 | 0 | 0 | 13.80 | 4.36 | 8.74 | 14000000 | 18127174.30 | 48827223.90 | 1 | 6.83 | 8 | 0.52 |
| *Sterna hirundo* | 0.9538 | -0.0531 | 0 | 1 | 2 | 0 | 125.00 | 2.65 | 2.65 | 420000 | 22446773.90 | 137032125.00 | 3 | 52.75 | 0 | 1.78 |
| *Streptopelia decaocto* | 0.9257 | 0.0772 | 0 | 0 | 0 | 1 | 201.50 | 1.97 | 11.84 | 7850000 | 18103545.30 | 84393908.40 | 4 | 0.00 | 0 | 1.35 |
| *Streptopelia turtur* | 0.8618 | -0.0300 | 0 | 0 | 0 | 0 | 136.50 | 1.90 | 5.70 | 5350000 | 17895086.00 | 49092130.50 | 4 | 25.90 | 2 | 1.27 |
| *Strix aluco* | 0.8958 | -0.0072 | 0 | 0 | 0 | 1 | 491.00 | 2.70 | 2.70 | 740000 | 16474024.50 | 55876079.20 | 11 | - | 3 | 9.57 |
| *Sturnus unicolor* | 0.7362 | 0.0301 | 1 | 1 | 0 | 1 | 90.60 | 4.10 | 8.20 | 2600000 | 2134572.98 | 3716312.32 | 1 | 0.00 | - | 1.83 |
| *Sturnus vulgaris* | 0.9144 | 0.0173 | 1 | 1 | 0 | 1 | 80.50 | 5.40 | 10.80 | 39500000 | 21868439.40 | 57229673.20 | 12 | 2.63 | 2 | 1.71 |
| *Sylvia atricapilla* | 0.8505 | 0.0331 | 1 | 0 | 0 | 1 | 18.85 | 4.65 | 9.30 | 37000000 | 22047377.30 | 49004643.50 | 5 | 19.64 | 3 | 0.63 |
| *Sylvia borin* | 0.8604 | -0.0233 | 0 | 0 | 0 | 0 | 19.05 | 4.03 | 8.06 | 24000000 | 16642794.90 | 24656981.80 | 2 | 63.25 | 5 | 0.58 |
| *Sylvia cantillans* | 0.9211 | 0.0413 | 1 | 0 | 0 | 0 | 8.10 | 4.20 | 8.40 | 2300000 | 4154768.05 | 7240806.32 | 3 | 20.82 | 1 | - |
| *Sylvia communis* | 0.8879 | -0.0006 | 1 | 0 | 0 | 0 | 14.50 | 4.63 | 9.26 | 19500000 | 18087278.10 | 34153491.30 | 4 | 53.05 | 3 | 0.52 |
| *Sylvia curruca* | 0.9408 | 0.0135 | 0 | 0 | 0 | 1 | 12.40 | 4.78 | 9.56 | 6300000 | 16741079.00 | 44818320.60 | 9 | 27.79 | 4 | 0.51 |
| *Sylvia hortensis* | 0.9557 | 0.1177 | 1 | 0 | 0 | 0 | 22.55 | 4.20 | 4.20 | 325000 | 8147539.36 | 16088720.30 | 3 | 15.02 | 1 | 0.68 |
| *Sylvia melanocephala* | 0.8275 | 0.0145 | 1 | 0 | 0 | 0 | 13.45 | 4.20 | 8.40 | 5600000 | 5640381.14 | 12182596.70 | 4 | 3.61 | 0 | 0.52 |
| *Sylvia nisoria* | 0.9747 | 0.0987 | 1 | 0 | 0 | 0 | 24.35 | 5.02 | 5.02 | 730000 | 9736736.71 | 17364578.10 | 2 | 44.78 | 0 | - |
| *Sylvia undata* | 0.8923 | -0.0345 | 1 | 0 | 0 | 0 | 8.94 | 4.00 | 12.00 | 2800000 | 4031074.21 | 4095074.62 | 3 | 3.52 | - | - |
| *Tachybaptus ruficollis* | 1.1379 | -0.0279 | 0 | 0 | 2 | 0 | 190.00 | 5.00 | 15.00 | 134500 | 15804739.70 | 129888352.00 | 9 | - | - | 1.62 |
| *Tachymarptis melba* | 1.0784 | -0.0546 | 0 | 1 | 0 | 1 | 92.00 | 2.70 | 2.70 | 235000 | 8360544.39 | 87113999.50 | 10 | 11.52 | 0 | 1.13 |
| *Tadorna tadorna* | 0.9745 | 0.0290 | 1 | 0 | 2 | 0 | 1152.00 | 8.99 | 9.00 | 53500 | 17039170.60 | 38534753.90 | 1 | 9.35 | 0 | 5.55 |
| *Tetrastes bonasia* | 0.9274 | -0.0111 | 1 | 0 | 0 | 0 | 369.50 | 9.38 | 9.38 | 2800000 | 12550567.50 | 35793103.00 | 4 | 0.00 | 2 | 1.88 |
| *Tetrax tetrax* | 0.9018 | 0.0318 | 1 | 0 | 0 | 0 | 854.50 | 3.50 | 3.50 | 210000 | 9898170.96 | 13465079.40 | 1 | - | 2 | - |
| *Tringa glareola* | 0.9778 | 0.0188 | 0 | 0 | 1 | 0 | 67.50 | 4.00 | 4.00 | 775000 | 8075508.15 | 22194410.90 | 1 | 62.89 | 0 | 1.20 |
| *Tringa nebularia* | 0.9648 | 0.0022 | 0 | 0 | 1 | 0 | 173.50 | 3.91 | 3.91 | 117500 | 8960859.31 | 22863286.80 | 1 | 49.16 | 0 | 1.81 |
| *Tringa ochropus* | 0.9485 | 0.0299 | 0 | 0 | 1 | 0 | 84.80 | 4.00 | 4.00 | 565000 | 11014939.90 | 31097993.10 | 1 | 36.42 | 2 | 1.30 |
| *Tringa totanus* | 0.9680 | -0.0180 | 0 | 0 | 1 | 0 | 112.00 | 3.95 | 3.95 | 445000 | 22229241.30 | 52792516.10 | 6 | 35.28 | 0 | 1.35 |
| *Troglodytes troglodytes* | 0.9540 | 0.0064 | 0 | 0 | 0 | 1 | 8.90 | 5.70 | 11.40 | 31500000 | 21608445.70 | 85810285.00 | 42 | 1.34 | 5 | 0.47 |
| *Turdus iliacus* | 0.8387 | 0.0227 | 0 | 0 | 0 | 1 | 62.85 | 5.50 | 11.00 | 18500000 | 11446024.50 | 25824770.60 | 2 | 10.77 | 3 | 1.33 |
| *Turdus merula* | 0.7970 | 0.0000 | 1 | 0 | 0 | 1 | 95.85 | 3.87 | 15.48 | 61000000 | 22419730.10 | 86850440.30 | 15 | 3.98 | 12 | 1.93 |
| *Turdus philomelos* | 0.8604 | 0.0244 | 0 | 0 | 0 | 1 | 70.50 | 4.70 | 18.80 | 28000000 | 17678168.00 | 30851068.70 | 4 | 14.65 | 4 | 1.59 |
| *Turdus pilaris* | 0.9873 | -0.0078 | 0 | 1 | 0 | 1 | 92.10 | 5.20 | 10.40 | 19000000 | 11244833.80 | 24506817.70 | 1 | 10.77 | 6 | 1.79 |
| *Turdus torquatus* | 1.0194 | 0.0568 | 1 | 0 | 0 | 0 | 117.00 | 4.10 | 8.20 | 490000 | 17540214.70 | 17793028.40 | 3 | 14.13 | 3 | 1.73 |
| *Turdus viscivorus* | 0.9632 | 0.0250 | 0 | 0 | 0 | 0 | 117.80 | 3.92 | 11.76 | 5200000 | 17984922.30 | 33724780.60 | 3 | 4.36 | 3 | 2.40 |
| *Upupa epops* | 1.0110 | -0.0505 | 1 | 0 | 0 | 0 | 67.05 | 7.00 | 14.00 | 1295000 | 16140429.70 | 136372138.00 | 9 | 9.59 | 2 | 1.05 |
| *Vanellus vanellus* | 0.9149 | -0.0015 | 1 | 0 | 1 | 0 | 218.50 | 3.85 | 3.85 | 2250000 | 17776382.30 | 37072931.00 | 1 | 12.08 | 2 | 2.06 |

S1 Table (continuation)

| Species | Distance to mainland  (km) | Annual adult survival  rate (%) | Flight initiation distance  (m) | CV in population  density | Change in first arrival  date (d/yr) | Change in mean arrival  date (d/yr) | Nest predation (%) | Susceptibility to  sparrowhawk predation | Susceptibility to  goshawk predation | Susceptibility to cat  predation | Natal dispersal (km) | Band-sharing coefficient | Number of alleles | Proportion of  polymorphic loci | Inbreeding coefficient |
| --- | --- | --- | --- | --- | --- | --- | --- | --- | --- | --- | --- | --- | --- | --- | --- |
| *Acanthis flammea* | 390 | 42.50 | 4.50 | 1.3510 | -0.20 | -0.1947 | 65.50 | -0.8997 | - | - | 2.14 | 0.100 | - | - | - |
| *Accipiter gentilis* | - | 70.00 | - | - | - | - | - | - | 0.5703 | - | 18.84 | - | 4.55 | 0.9167 | 0.1360 |
| *Accipiter nisus* | - | 43.00 | 10.00 | - | -1.88 | -0.0489 | - | -1.3667 | 0.0323 | - | 6.65 | - | - | - | - |
| *Acrocephalus arundinaceus* | 110 | 65.00 | - | 2.3796 | -0.11 | - | - | - | - | - | - | 0.240 | 9.54 | 0.9231 | - |
| *Acrocephalus palustris* | 40 | 49.25 | 8.84 | 1.3833 | -0.42 | -0.1592 | 15.30 | -0.4737 | - | - | - | 0.200 | - | - | - |
| *Acrocephalus schoenobaenus* | 40 | 31.00 | 7.56 | 1.3580 | -0.19 | -0.0600 | - | - | - | - | 6.02 | 0.267 | - | - | - |
| *Acrocephalus scirpaceus* | 60 | 56.00 | 6.73 | 2.3627 | -0.31 | -0.1554 | 20.00 | - | - | 0.0000 | 5.22 | - | - | - | - |
| *Actitis hypoleucos* | - | 75.00 | 17.00 | - | -0.22 | 0.1172 | 6.70 | -0.0758 | -0.0680 | 0.0000 | - | 0.091 | - | - | - |
| *Aegithalos caudatus* | 60 | 55.00 | 5.02 | 1.1370 | - | - | - | -0.6778 | - | - | 2.18 | - | - | - | - |
| *Alauda arvensis* | 310 | 66.50 | 31.37 | 1.1119 | -0.48 | -0.3075 | - | -0.1424 | -0.9613 | -0.9379 | 0.30 | - | 8.13 | - | - |
| *Alcedo atthis* | - | 23.80 | 16.27 | - | - | - | - | - | - | - | 3.18 | - | - | - | - |
| *Alectoris rufa* | - | - | 35.54 | - | - | - | - | - | - | - | - | - | 8.00 | 1.0000 | 0.0946 |
| *Anas clypeata* | - | 56.00 | - | - | -1.26 | -0.0406 | 42.00 | - | - | - | - | - | - | - | - |
| *Anas crecca* | 400 | 46.00 | 39.23 | - | -0.59 | -0.1413 | - | - | 1.8958 | - | - | - | - | - | - |
| *Anas platyrhynchos* | 2730 | 52.00 | 13.42 | - | -0.31 | -0.4782 | 16.40 | - | 0.2842 | - | - | - | 5.80 | 0.9600 | 0.2828 |
| *Anas querquedula* | - | - | - | - | -0.63 | - | - | - | - | - | - | - | - | - | - |
| *Anas strepera* | - | - | - | - | -0.88 | - | 55.00 | - | - | - | - | - | 8.67 | 1.0000 | 0.0860 |
| *Anser anser* | 400 | 77.00 | - | - | -0.76 | 0.1189 | - | - | -0.5865 | - | - | - | - | - | - |
| *Anthus campestris* | 80 | - | 18.20 | 2.5268 | - | - | - | -0.3768 | - | - | - | - | - | - | - |
| *Anthus pratensis* | 390 | 65.00 | 13.97 | 1.4857 | -0.44 | -0.3662 | 21.00 | 0.1855 | - | 0.0000 | - | - | - | - | - |
| *Anthus spinoletta* | 310 | - | 4.16 | 1.7058 | -0.22 | -0.3294 | 39.00 | - | - | - | - | 0.236 | - | - | - |
| *Anthus trivialis* | 60 | 42.85 | 10.19 | 1.1954 | -0.24 | -0.1711 | - | 0.1557 | -1.3690 | - | - | - | - | - | - |
| *Apus apus* | 68 | 84.50 | 38.10 | 0.9156 | -0.19 | -0.0655 | - | -1.5529 | - | 0.6021 | 11.47 | 0.177 | - | - | - |
| *Ardea cinerea* | 380 | 69.00 | 47.36 | - | -0.90 | - | - | - | -1.1399 | -0.3010 | 24.08 | - | - | - | - |
| *Ardea purpurea* | 380 | - | - | - | - | - | - | - | - | - | - | - | - | - | - |
| *Asio otus* | 1360 | 69.00 | - | 0.9156 | - | - | - | 0.2552 | 2.3814 | - | - | 0.250 | - | - | - |
| *Athene noctua* | 68 | 64.80 | 36.43 | 1.0895 | - | - | 5.30 | - | 1.8862 | - | - | 0.220 | - | - | - |
| *Aythya ferina* | - | - | - | - | -0.65 | - | 21.62 | - | - | - | - | - | - | - | - |
| *Aythya fuligula* | - | 54.00 | 10.68 | - | -0.53 | -0.0728 | 38.00 | - | 0.2050 | - | - | - | - | - | - |
| *Branta leucopsis* | - | - | - | 2.4207 | -1.31 | - | - | - | - | - | - | 0.280 | - | - | - |
| *Bubulcus ibis* | - | 75.00 | 24.38 | - | - | - | - | - | - | - | - | - | - | - | - |
| *Bucephala clangula* | - | 63.00 | - | - | -0.93 | -0.1705 | - | - | - | - | - | - | - | - | - |
| *Burhinus oedicnemus* | - | - | - | - | - | - | 10.20 | - | - | - | - | - | - | - | - |
| *Buteo buteo* | - | 80.90 | 54.06 | - | -0.92 | - | - | - | -0.8671 | -0.9031 | 12.10 | - | 4.46 | 1.0000 | -0.0075 |
| *Calandrella brachydactyla* | 80 | - | - | 3.2286 | - | - | - | - | - | - | - | - | - | - | - |
| *Carduelis carduelis* | 1360 | 35.00 | 7.63 | 0.8087 | -0.30 | -0.3912 | 75.00 | -0.2629 | -0.8919 | 0.3010 | 1.66 | - | - | - | - |
| *Carduelis citrinella* | 60 | 54.00 | - | 2.6579 | - | - | - | - | - | - | - | - | - | - | - |
| *Carpodacus erythrinus* | 40 | 76.00 | - | 2.1367 | -1.19 | 0.2213 | 37.00 | - | - | - | - | - | - | - | - |
| *Cecropis daurica* | 80 | - | 8.06 | 2.2329 | - | - | - | - | - | - | - | - | - | - | - |
| *Certhia brachydactyla* | 60 | - | 7.08 | 1.4732 | - | - | - | - | - | - | - | - | - | - | - |
| *Certhia familiaris* | 60 | 44.00 | 4.47 | 0.8905 | - | - | 68.10 | 0.7693 | -0.0680 | - | - | - | - | - | - |
| *Cettia cetti* | 60 | - | 5.55 | 1.9643 | - | - | - | - | - | - | - | - | - | - | - |
| *Charadrius dubius* | - | 45.00 | 9.00 | - | -0.81 | - | - | - | - | - | - | - | - | - | - |
| *Chloris chloris* | 1360 | 43.00 | 6.59 | 0.7812 | 0.00 | -0.2993 | 40.80 | 0.1759 | -1.0266 | -0.6021 | 0.95 | - | - | - | - |
| *Chroicocephalus ridibundus* | - | 76.00 | 41.20 | - | -0.41 | 0.1121 | - | - | 0.3432 | -0.6990 | 10.53 | - | - | - | - |
| *Ciconia ciconia* | 10 | 79.00 | 35.00 | - | -0.17 | - | - | - | - | - | - | - | - | - | - |
| *Cinclus cinclus* | 60 | 57.50 | - | 1.1050 | - | - | 18.40 | - | - | - | 1.68 | - | - | - | - |
| *Circaetus gallicus* | - | - | - | - | - | - | - | - | - | - | - | - | - | - | - |
| *Circus aeruginosus* | - | 74.00 | - | - | 0.12 | - | - | - | - | - | - | - | - | - | - |
| *Circus cyaneus* | - | 72.40 | - | - | 0.12 | - | - | - | - | - | 10.73 | - | - | - | - |
| *Circus pygargus* | - | - | - | - | - | - | 1.00 | - | - | - | - | - | - | - | - |
| *Cisticola juncidis* | 110 | - | 5.32 | 3.1032 | - | - | 21.00 | - | - | - | - | - | - | - | - |
| *Clamator glandarius* | - | - | - | - | - | - | - | - | - | - | - | - | 6.71 | 1.0000 | -0.0183 |
| *Coccothraustes coccothraustes* | 40 | - | 8.60 | 1.4585 | -0.97 | - | 54.00 | 0.5489 | 0.8273 | - | - | - | - | - | - |
| *Coloeus monedula* | 60 | 65.00 | 26.67 | 1.6564 | -0.52 | -0.8333 | - | -2.0758 | -0.5430 | - | 2.13 | 0.085 | - | - | - |
| *Columba livia* | 1360 | 66.50 | 6.46 | - | - | - | 26.80 | - | - | 0.0000 | - | - | 4.61 | 0.9821 | - |
| *Columba oenas* | 50 | 55.00 | 5.00 | - | -0.78 | -0.9710 | 19.00 | - | -1.5561 | - | 3.04 | - | - | - | - |
| *Columba palumbus* | - | 64.00 | 19.69 | - | -0.77 | -0.3765 | - | -1.1579 | 0.5007 | -0.3010 | 2.28 | - | - | - | - |
| *Coracias garrulus* | - | - | - | - | - | - | - | - | - | - | - | - | - | - | - |
| *Corvus corax* | 390 | - | 79.63 | 0.8476 | - | - | - | - | -0.5451 | - | 25.84 | - | - | - | - |
| *Corvus corone* | 60 | 73.60 | 27.48 | 0.8140 | -0.73 | -0.2319 | 68.70 | - | -0.2019 | 0.0000 | 3.24 | - | 8.14 | 1.0000 | -0.0537 |
| *Corvus frugilegus* | 50 | 62.00 | 14.39 | 1.8619 | -0.52 | -0.1479 | - | - | -0.4552 | -0.6021 | 1.96 | - | 16.29 | 1.0000 | 0.0719 |
| *Coturnix coturnix* | 1360 | - | - | 1.7780 | -0.73 | - | - | 0.6232 | - | - | - | 0.210 | - | - | - |
| *Crex crex* | - | - | - | - | -0.52 | - | - | - | - | - | - | - | - | - | - |
| *Cuculus canorus* | 68 | - | 25.89 | 0.6033 | -0.12 | 0.2488 | - | -1.2307 | -0.7256 | -0.3010 | - | 0.210 | 7.94 | 1.0000 | -0.2307 |
| *Cyanistes caeruleus* | 110 | 41.60 | 5.54 | 1.2150 | - | -0.1198 | 19.10 | 0.0351 | -2.1094 | 0.6021 | 0.80 | 0.194 | 14.49 | 0.9714 | 0.0102 |
| *Cyanopica cyanus* | 10 | - | - | 3.4638 | - | - | 62.00 | - | - | - | - | 0.522 | - | - | - |
| *Cygnus cygnus* | - | 83.00 | 155.00 | - | -0.20 | -0.4910 | - | - | - | - | - | - | - | - | - |
| *Cygnus olor* | - | 81.00 | - | - | -0.97 | -0.3421 | - | - | - | - | 16.77 | - | - | - | - |
| *Delichon urbicum* | 130 | 43.00 | 6.34 | 0.7002 | -1.21 | 0.0095 | 12.00 | -0.5281 | - | -0.7782 | 3.19 | 0.195 | - | - | - |
| *Dendrocopos major* | 75 | 57.40 | 13.44 | 0.7659 | - | - | - | 0.3454 | 0.5804 | -0.3010 | 5.88 | 0.350 | - | - | - |
| *Dendrocopos medius* | 0 | 69.20 | - | 1.2550 | - | - | - | - | - | - | - | 0.480 | - | - | - |
| *Dendrocopos minor* | - | 61.00 | - | - | - | - | - | - | - | - | - | - | - | - | - |
| *Dendrocopos syriacus* | - | - | - | - | - | - | - | - | - | - | - | - | - | - | - |
| *Dryocopus martius* | - | 70.10 | 38.71 | - | - | - | - | - | - | - | - | - | - | - | - |
| *Egretta garzetta* | - | - | 24.50 | - | - | - | - | - | - | - | - | - | - | - | - |
| *Emberiza calandra* | 110 | 48.00 | 9.13 | 1.8297 | - | - | - | -0.6216 | -1.3934 | - | - | 0.180 | - | - | - |
| *Emberiza cia* | 40 | - | 9.43 | 3.2236 | - | - | 48.70 | - | - | - | - | - | - | - | - |
| *Emberiza cirlus* | 40 | - | 7.00 | 1.5645 | - | - | - | - | - | - | - | - | - | - | - |
| *Emberiza citrinella* | 40 | 53.00 | 9.99 | 1.2281 | -0.34 | -0.2137 | 36.90 | 0.0609 | -1.2492 | -1.1139 | 2.03 | - | 7.91 | 1.0000 | -0.0100 |
| *Emberiza hortulana* | 40 | - | - | 1.3011 | -0.86 | 0.0455 | 45.90 | - | - | - | - | - | - | - | - |
| *Emberiza rustica* | 30 | - | - | 3.1213 | -0.22 | - | 20.50 | - | - | - | - | - | - | - | - |
| *Emberiza schoeniclus* | 40 | 51.70 | 9.81 | 0.7908 | -0.51 | -0.2499 | 59.30 | -0.1865 | -1.6701 | - | 0.95 | - | 12.93 | 0.9333 | 0.1009 |
| *Erithacus rubecula* | 1360 | 38.00 | 5.13 | 0.6332 | -0.54 | -0.2965 | - | 0.1760 | -1.0100 | 0.3680 | 0.57 | - | - | - | - |
| *Falco naumanni* | 28 | - | - | 2.0813 | - | - | - | - | - | - | - | 0.216 | - | - | - |
| *Falco peregrinus* | 380 | 75.00 | - | - | -0.13 | - | - | - | - | - | 13.50 | - | 3.53 | 0.8421 | -0.2151 |
| *Falco subbuteo* | - | - | - | - | -0.26 | - | - | - | - | - | - | - | 3.06 | 0.8750 | -0.1356 |
| *Falco tinnunculus* | - | 65.00 | 30.94 | - | -0.13 | - | 23.00 | -1.7747 | 0.3470 | - | 14.01 | - | 2.25 | 0.7500 | -0.4707 |
| *Ficedula albicollis* | 60 | 45.00 | - | 2.2411 | 0.05 | - | 13.00 | - | - | - | - | - | 12.33 | 1.0000 | -0.0161 |
| *Ficedula hypoleuca* | 40 | 49.90 | 5.39 | 1.3887 | -0.19 | -0.1528 | - | 0.6374 | - | - | 14.27 | 0.193 | 12.62 | 1.0000 | 0.0560 |
| *Fringilla coelebs* | 1360 | 64.00 | 7.14 | 0.4045 | -0.31 | -0.1548 | - | 0.0632 | -0.8074 | -0.6021 | 0.79 | 0.162 | - | - | - |
| *Fringilla montifringilla* | 10 | - | - | 2.0875 | -0.50 | -0.1519 | - | - | - | - | - | - | - | - | - |
| *Fulica atra* | 1500 | 76.50 | 18.57 | - | -0.32 | - | 16.00 | -1.9209 | -0.0210 | - | 4.44 | - | - | - | - |
| *Galerida cristata* | 60 | - | 14.33 | 1.5257 | - | - | - | -0.0758 | - | - | - | - | - | - | - |
| *Galerida theklae* | 80 | - | 7.18 | 3.4641 | - | - | - | - | - | - | - | - | - | - | - |
| *Gallinago gallinago* | - | 48.10 | 25.83 | - | -0.26 | -0.0081 | - | 0.2207 | 0.5295 | - | - | - | - | - | - |
| *Gallinula chloropus* | 1000 | - | 9.88 | 1.4463 | -0.51 | - | 8.00 | -2.3591 | -0.1260 | - | 0.56 | 0.266 | - | - | - |
| *Garrulus glandarius* | 60 | 59.00 | 11.76 | 1.4506 | - | - | 54.00 | 0.4557 | 1.6341 | - | 0.70 | - | - | - | - |
| *Gavia arctica* | - | 89.00 | - | - | -0.41 | 0.2342 | - | - | - | - | - | - | - | - | - |
| *Gavia stellata* | - | - | - | - | -0.58 | - | - | - | - | - | - | - | - | - | - |
| *Grus grus* | - | - | 108.00 | - | -0.94 | - | - | - | - | - | - | - | - | - | - |
| *Gyps fulvus* | - | - | - | - | - | - | - | - | - | - | - | - | 5.00 | 1.0000 | -0.0123 |
| *Haematopus ostralegus* | 400 | 84.10 | - | 1.4463 | -0.63 | -0.0501 | 20.00 | - | - | - | - | 0.220 | 3.28 | 1.0000 | 0.0527 |
| *Himantopus himantopus* | - | - | 22.00 | - | - | - | - | - | - | - | - | - | - | - | - |
| *Hippolais icterina* | 60 | 34.80 | 7.52 | 1.7399 | -0.60 | -0.0675 | - | -1.6778 | - | -0.8451 | - | - | - | - | - |
| *Hippolais polyglotta* | 60 | - | - | 2.6141 | - | - | - | - | - | - | - | - | - | - | - |
| *Hirundo rustica* | 130 | 37.00 | 10.16 | 0.7186 | -0.09 | -0.2051 | 5.00 | -0.1105 | -2.2643 | -1.6021 | 3.19 | 0.106 | 21.27 | 1.0000 | 0.0037 |
| *Ichthyaetus melanocephalus* | - | - | - | - | - | - | - | - | - | - | - | - | - | - | - |
| *Jynx torquilla* | - | - | - | - | -0.32 | 0.1239 | - | 1.2253 | - | - | - | - | - | - | - |
| *Lagopus lagopus* | 68 | 35.00 | - | 2.0055 | - | - | 24.00 | - | - | - | - | 0.292 | 13.41 | 1.0000 | 0.1374 |
| *Lanius collurio* | 60 | 50.00 | 7.27 | 1.3878 | -0.03 | -0.0498 | 59.00 | 1.1146 | 0.4091 | 0.0000 | - | - | - | - | - |
| *Lanius excubitor* | - | - | 21.16 | 2.8903 | - | - | 33.00 | - | - | - | - | - | - | - | - |
| *Lanius meridionalis* | - | - | - | - | - | - | - | - | - | - | - | - | - | - | - |
| *Lanius minor* | 10 | - | - | 2.7967 | - | - | - | - | - | - | - | 0.390 | - | - | - |
| *Lanius senator* | 60 | - | 10.99 | 2.9076 | - | - | 21.00 | - | - | - | - | - | - | - | - |
| *Larus argentatus* | - | 93.50 | 40.15 | - | -0.07 | -0.5189 | - | - | -0.7959 | -0.3010 | - | - | - | - | - |
| *Larus canus* | - | 74.00 | 59.94 | 1.4828 | -0.61 | -0.3113 | - | - | 0.1718 | -0.9031 | 4.73 | 0.190 | - | - | - |
| *Larus fuscus* | 400 | 91.00 | 37.00 | - | -0.75 | -0.3496 | - | - | - | -0.3010 | 2.38 | - | - | - | - |
| *Larus marinus* | - | - | 68.00 | - | -0.16 | -0.5093 | - | - | - | - | - | - | - | - | - |
| *Larus michahellis* | - | - | - | - | - | - | - | - | - | - | - | - | - | - | - |
| *Limosa limosa* | - | 63.10 | - | - | -0.29 | - | 21.50 | - | - | - | - | - | - | - | - |
| *Linaria cannabina* | 110 | 34.00 | 9.25 | 0.8674 | -0.13 | 0.1449 | 40.80 | 0.4034 | -1.7212 | -0.7782 | 0.69 | - | - | - | - |
| *Locustella fluviatilis* | 40 | - | - | 1.9769 | 0.01 | - | 43.00 | - | - | - | - | - | - | - | - |
| *Locustella luscinioides* | 40 | - | - | 2.8196 | -0.29 | - | 27.40 | - | - | - | - | - | - | - | - |
| *Locustella naevia* | 40 | - | 15.03 | 1.5722 | -0.09 | - | - | - | - | - | - | - | - | - | - |
| *Lophophanes cristatus* | 40 | 53.70 | 6.32 | 1.1945 | - | - | 39.00 | -0.0471 | - | - | - | - | - | - | - |
| *Loxia curvirostra* | 110 | - | 4.74 | 0.9898 | - | - | - | 1.3714 | 0.8351 | - | - | - | - | - | - |
| *Lullula arborea* | 60 | - | 12.14 | 2.6094 | -0.29 | - | - | 0.2253 | -0.0680 | - | - | - | - | - | - |
| *Luscinia luscinia* | 60 | - | 15.89 | 1.7290 | -0.31 | -0.1247 | - | -2.4182 | - | 0.0000 | - | - | - | - | - |
| *Luscinia megarhynchos* | 60 | 48.50 | 8.16 | 1.2149 | -0.22 | - | 7.50 | - | - | - | - | - | - | - | - |
| *Luscinia svecica* | 10 | 66.00 | - | 2.0239 | -0.07 | -0.0930 | - | - | - | - | - | 0.290 | 11.00 | 1.0000 | - |
| *Lyrurus tetrix* | 38 | 50.00 | - | 1.5289 | - | - | - | - | - | - | - | 0.110 | 10.57 | 1.0000 | 0.0156 |
| *Melanocorypha calandra* | 60 | - | - | 3.3220 | - | - | - | - | - | - | - | - | - | - | - |
| *Mergus merganser* | - | 60.00 | - | - | -0.83 | -0.1469 | - | - | - | - | - | - | 6.89 | 1.0000 | 0.2549 |
| *Mergus serrator* | - | - | - | - | -0.73 | -0.2250 | 35.00 | - | - | - | - | - | - | - | - |
| *Merops apiaster* | 68 | - | 37.02 | 1.7359 | - | - | - | - | - | - | - | 0.189 | 5.75 | 1.0000 | -0.0088 |
| *Milvus migrans* | - | - | 37.91 | - | -0.77 | - | - | - | - | - | - | - | - | - | - |
| *Milvus milvus* | - | 82.20 | - | - | - | - | - | - | 0.9320 | - | - | - | - | - | - |
| *Monticola saxatilis* | 80 | - | 16.25 | 2.4230 | - | - | - | - | - | - | - | - | - | - | - |
| *Monticola solitarius* | 130 | - | - | 1.8270 | - | - | - | - | - | - | - | - | - | - | - |
| *Motacilla alba* | 390 | 52.00 | 11.32 | 0.8177 | -0.76 | -0.0964 | 23.60 | 0.2698 | -1.2643 | -0.6990 | 5.16 | - | - | - | - |
| *Motacilla cinerea* | 1360 | 39.00 | 7.07 | 1.4504 | - | - | - | 0.2253 | - | - | 15.80 | - | - | - | - |
| *Motacilla flava* | 60 | 34.25 | 11.24 | 1.1788 | -0.29 | -0.0308 | 29.20 | 0.9454 | - | - | 4.95 | - | - | - | - |
| *Muscicapa striata* | 40 | 72.00 | 8.50 | 1.3183 | -0.12 | -0.0929 | 13.20 | -0.1080 | -0.9131 | 0.3010 | 3.38 | - | - | - | - |
| *Nucifraga caryocatactes* | 130 | - | - | 1.2199 | - | - | - | - | - | - | - | - | - | - | - |
| *Numenius arquata* | - | 73.60 | 62.75 | - | -0.16 | 0.0059 | 5.71 | - | 0.2330 | - | - | - | - | - | - |
| *Numenius phaeopus* | - | 69.20 | - | - | -0.22 | -0.1792 | - | - | - | - | - | - | - | - | - |
| *Nycticorax nycticorax* | - | 75.45 | - | - | - | - | - | - | - | - | - | - | - | - | - |
| *Oenanthe hispanica* | 40 | - | - | 2.7272 | - | - | - | - | - | - | - | - | - | - | - |
| *Oenanthe leucura* | 0 | - | 30.37 | 3.4737 | - | - | 41.20 | - | - | - | - | - | - | - | - |
| *Oenanthe oenanthe* | 390 | 55.00 | 15.80 | 1.6551 | -0.54 | 0.0042 | 28.00 | 0.7217 | -0.8084 | - | 2.35 | 0.210 | - | - | - |
| *Oriolus oriolus* | 60 | - | 39.67 | 1.2351 | -0.21 | - | 14.30 | - | - | - | - | - | - | - | - |
| *Pandion haliaetus* | - | 81.50 | - | - | -0.08 | - | - | - | - | - | - | - | - | - | - |
| *Parus major* | 130 | 48.60 | 5.22 | 0.9591 | -0.29 | -0.2627 | 23.30 | 0.3254 | -1.2676 | -0.8846 | 0.80 | 0.215 | 10.70 | 1.0000 | 0.0163 |
| *Passer domesticus* | 60 | 55.00 | 3.83 | 0.6618 | - | - | - | 0.2555 | -2.0222 | -1.8162 | 0.21 | 0.097 | 15.50 | 1.0000 | -0.0095 |
| *Passer montanus* | 130 | 41.50 | 5.11 | 0.7131 | - | - | - | 0.7293 | -1.8742 | -0.8653 | 1.06 | - | - | - | - |
| *Perdix perdix* | - | 44.00 | 24.78 | - | - | - | 26.00 | -0.5987 | 1.6310 | - | - | - | - | - | - |
| *Periparus ater* | 130 | 33.80 | 5.55 | 0.7441 | - | - | - | -0.4356 | - | - | 2.60 | 0.170 | - | - | - |
| *Perisoreus infaustus* | 0 | - | - | 2.0404 | - | - | 80.00 | - | - | - | - | 0.430 | 6.89 | 1.0000 | -0.0437 |
| *Pernis apivorus* | - | - | - | - | -0.34 | - | - | - | 1.7101 | - | - | - | - | - | - |
| *Petronia petronia* | 110 | - | - | 3.2183 | - | - | - | - | - | - | - | 0.220 | - | - | - |
| *Phalacrocorax carbo* | - | 89.50 | 75.29 | - | -0.51 | -0.3295 | - | - | - | - | - | - | 27.71 | 1.0000 | - |
| *Philomachus pugnax* | - | 52.40 | - | - | -0.14 | 0.0024 | 24.14 | - | - | - | - | - | 19.11 | 1.0000 | 0.1196 |
| *Phoenicurus ochruros* | 40 | 55.30 | 6.95 | 1.3236 | -0.08 | -0.3239 | - | -0.0758 | - | 0.0000 | - | - | - | - | - |
| *Phoenicurus phoenicurus* | 40 | 38.00 | 9.10 | 0.6545 | -0.44 | -0.1032 | - | -0.4737 | - | 0.6990 | 3.87 | - | - | - | - |
| *Phylloscopus bonelli* | 60 | - | 4.95 | 2.5983 | - | - | - | - | - | - | - | - | - | - | - |
| *Phylloscopus collybita* | 110 | 36.70 | 6.49 | 0.6795 | -0.17 | -0.2227 | 17.00 | - | - | -0.7782 | - | - | - | - | - |
| *Phylloscopus sibilatrix* | 40 | 38.60 | - | 2.3465 | -0.13 | 0.2491 | - | -1.1550 | - | - | - | - | - | - | - |
| *Phylloscopus trochiloides* | 60 | - | - | 3.0098 | -0.36 | - | 20.00 | - | - | - | - | - | - | - | - |
| *Phylloscopus trochilus* | 40 | 32.70 | 6.28 | 1.1636 | -0.38 | -0.0875 | 30.00 | -0.5934 | - | -0.4771 | 2.17 | 0.150 | 4.67 | 1.0000 | 0.0608 |
| *Pica pica* | 130 | 69.00 | 14.37 | 1.0286 | - | - | - | -1.6778 | 0.7355 | 0.0000 | 1.03 | 0.155 | 13.67 | 1.0000 | 0.1319 |
| *Picus canus* | 120 | - | - | - | - | - | - | - | - | - | - | - | - | - | - |
| *Picus viridis* | 100 | - | 17.00 | - | - | - | - | 0.4983 | 2.0112 | - | 2.05 | - | - | - | - |
| *Pluvialis apricaria* | - | 61.00 | 47.00 | - | -0.67 | -0.4790 | 10.60 | - | - | - | - | - | - | - | - |
| *Podiceps auritus* | - | - | - | - | -0.37 | - | 21.30 | - | - | - | - | - | - | - | - |
| *Podiceps cristatus* | - | - | - | - | -0.45 | -0.1146 | - | - | - | - | - | - | - | - | - |
| *Podiceps grisegena* | - | 80.50 | - | - | -0.29 | -0.0768 | - | - | - | - | - | - | 11.00 | 1.0000 | 0.1393 |
| *Poecile montanus* | 40 | 60.00 | - | 1.1292 | - | - | 61.00 | - | - | - | 1.06 | 0.200 | - | - | - |
| *Poecile palustris* | 40 | - | 5.59 | 0.9760 | - | - | 7.10 | -0.4163 | - | -0.6990 | - | - | - | - | - |
| *Prunella collaris* | 130 | 50.80 | - | 1.7227 | - | - | - | - | - | - | - | 0.136 | 2.78 | 1.0000 | -0.0269 |
| *Prunella modularis* | 40 | 49.00 | 5.08 | 1.1118 | -0.02 | -0.1702 | - | 0.3308 | -1.1929 | 0.6021 | 0.38 | 0.240 | - | - | - |
| *Ptyonoprogne rupestris* | 80 | - | - | 2.3674 | - | - | - | - | - | - | - | - | - | - | - |
| *Pyrrhocorax graculus* | 60 | - | - | 1.5539 | - | - | - | - | - | - | - | - | - | - | - |
| *Pyrrhocorax pyrrhocorax* | 110 | 74.00 | 120.00 | 1.8140 | - | - | - | - | - | - | - | - | - | - | - |
| *Pyrrhula pyrrhula* | 40 | 44.50 | 6.01 | 0.3980 | -0.29 | -0.3232 | - | 0.2600 | -0.3021 | 0.1761 | 0.85 | - | - | - | - |
| *Rallus aquaticus* | - | - | - | - | -0.58 | - | - | -1.1621 | -0.5523 | - | - | - | - | - | - |
| *Recurvirostra avosetta* | 50 | 71.80 | - | - | -0.44 | - | - | - | - | - | - | - | - | - | - |
| *Regulus ignicapilla* | 80 | - | 5.25 | 1.4592 | - | - | - | - | - | - | - | - | - | - | - |
| *Regulus regulus* | 1360 | 22.00 | 4.49 | 0.6716 | -1.37 | -0.1735 | 36.00 | -0.3434 | - | 0.4771 | - | - | - | - | - |
| *Remiz pendulinus* | 40 | - | 6.17 | 1.1529 | -0.71 | - | 4.00 | - | - | - | - | 0.390 | - | - | - |
| *Riparia riparia* | 80 | 35.00 | 23.10 | 0.7459 | 0.44 | - | 17.50 | -0.3267 | -2.0222 | - | 6.65 | 0.196 | - | - | - |
| *Saxicola rubetra* | 60 | 46.00 | 16.65 | 1.3849 | -0.40 | -0.0195 | 56.00 | 0.1997 | - | - | - | - | - | - | - |
| *Saxicola torquatus* | 400 | 31.90 | 9.27 | 1.1972 | - | - | 3.30 | - | - | - | - | - | - | - | - |
| *Scolopax rusticola* | - | 61.00 | 11.00 | - | -0.05 | -0.1844 | 15.00 | 0.9242 | 1.5193 | - | - | - | - | - | - |
| *Serinus serinus* | 110 | 60.30 | 5.84 | 1.7872 | -0.69 | - | - | - | - | - | - | 0.350 | - | - | - |
| *Sitta europaea* | 130 | 43.00 | 6.59 | 0.8981 | - | - | - | 1.2253 | 0.7771 | - | 1.80 | - | 9.80 | 1.0000 | 0.0221 |
| *Somateria mollissima* | - | 94.00 | - | - | -0.29 | -0.0475 | 20.30 | - | -1.3580 | - | - | - | 6.50 | 1.0000 | 0.2081 |
| *Spinus spinus* | 40 | 39.00 | 4.83 | 1.2561 | -0.58 | -0.2697 | - | 1.4865 | - | -1.4723 | - | - | - | - | - |
| *Sterna hirundo* | - | 92.00 | - | - | -0.58 | 0.6049 | - | - | - | - | - | - | - | - | - |
| *Streptopelia decaocto* | 275 | 61.00 | 5.38 | - | - | - | - | -0.7839 | 0.3792 | -0.3010 | 2.50 | - | - | - | - |
| *Streptopelia turtur* | 75 | 50.00 | - | - | -0.42 | - | 34.00 | - | - | - | 1.30 | - | - | - | - |
| *Strix aluco* | 120 | 52.40 | - | - | - | - | - | - | 1.3102 | 0.0000 | 2.12 | - | - | - | - |
| *Sturnus unicolor* | 60 | 49.90 | 14.30 | 3.0104 | - | - | - | - | - | - | - | 0.195 | - | - | - |
| *Sturnus vulgaris* | 1360 | 47.00 | 9.75 | 0.8600 | -0.01 | 0.0678 | - | 0.0388 | 0.1474 | -1.3979 | 1.10 | 0.143 | 14.00 | 1.0000 | -0.0500 |
| *Sylvia atricapilla* | 1360 | 46.00 | 5.84 | 0.7112 | -0.36 | -0.1997 | 21.90 | -0.4359 | - | 1.0000 | 17.54 | - | - | - | - |
| *Sylvia borin* | 40 | 45.70 | 6.60 | 0.8125 | -0.43 | -0.0188 | 21.60 | -0.3488 | -2.0222 | -0.3010 | - | - | - | - | - |
| *Sylvia cantillans* | 60 | - | 5.69 | 2.6320 | - | - | - | - | - | - | - | - | - | - | - |
| *Sylvia communis* | 40 | 26.10 | 8.15 | 0.6778 | -0.21 | -0.1365 | 21.40 | -0.2276 | -2.0680 | -1.0792 | 2.82 | - | - | - | - |
| *Sylvia curruca* | 40 | 45.80 | 5.28 | 0.7949 | -0.15 | -0.1527 | 26.00 | -0.0704 | - | -0.3979 | 16.89 | - | - | - | - |
| *Sylvia hortensis* | 40 | - | - | 3.0440 | - | - | - | - | - | - | - | - | - | - | - |
| *Sylvia melanocephala* | 110 | 55.00 | 6.64 | 1.7430 | - | - | - | - | - | - | - | - | - | - | - |
| *Sylvia nisoria* | 60 | 39.00 | - | 2.3241 | -0.12 | - | - | - | - | - | - | - | - | - | - |
| *Sylvia undata* | 80 | 50.00 | 10.16 | 2.9753 | - | - | - | - | - | - | - | - | - | - | - |
| *Tachybaptus ruficollis* | - | - | - | - | - | - | - | - | -0.2583 | - | - | - | - | - | - |
| *Tachymarptis melba* | - | 79.10 | - | - | - | - | - | - | - | - | - | - | - | - | - |
| *Tadorna tadorna* | - | 80.00 | 36.30 | - | -0.18 | -0.1349 | - | - | -0.7115 | - | - | - | - | - | - |
| *Tetrastes bonasia* | - | - | - | - | - | - | - | - | - | - | - | - | - | - | - |
| *Tetrax tetrax* | - | - | - | - | - | - | - | - | - | - | - | - | - | - | - |
| *Tringa glareola* | - | 53.60 | - | - | -0.25 | -0.0168 | - | - | - | - | - | - | - | - | - |
| *Tringa nebularia* | - | - | 30.00 | - | -0.20 | -0.2112 | - | - | - | - | - | - | - | - | - |
| *Tringa ochropus* | - | - | - | - | -0.29 | - | - | - | - | - | - | - | - | - | - |
| *Tringa totanus* | - | 71.30 | 29.71 | - | -0.35 | -0.0408 | 13.00 | -1.4182 | -0.6323 | - | - | - | - | - | - |
| *Troglodytes troglodytes* | 390 | 37.00 | 5.64 | 1.7563 | -0.27 | -0.2387 | - | -0.9886 | - | 0.4771 | 1.17 | - | - | - | - |
| *Turdus iliacus* | 390 | 42.50 | 12.75 | 1.8337 | -0.39 | -0.2162 | 32.00 | - | - | - | - | - | - | - | - |
| *Turdus merula* | 1360 | 56.00 | 7.11 | 0.9547 | -0.30 | -0.3255 | 18.00 | 0.2075 | 0.4366 | -0.1946 | 0.26 | 0.286 | - | - | - |
| *Turdus philomelos* | 40 | 46.00 | 7.73 | 0.6343 | -0.76 | -0.2116 | - | 0.8968 | 0.8947 | -0.6021 | 0.59 | - | - | - | - |
| *Turdus pilaris* | 40 | 35.00 | 14.21 | 1.2008 | -0.35 | -0.0130 | - | 2.2423 | 2.2416 | - | - | - | - | - | - |
| *Turdus torquatus* | 40 | - | - | 1.4579 | -0.57 | -0.0610 | 23.50 | - | - | 0.0000 | - | - | - | - | - |
| *Turdus viscivorus* | 40 | 52.00 | 19.33 | 0.6672 | -0.36 | -0.4323 | - | 0.1003 | 0.8809 | - | 1.49 | - | - | - | - |
| *Upupa epops* | 428 | 41.10 | 19.96 | 2.6631 | -0.67 | - | - | - | - | - | - | 0.180 | - | - | - |
| *Vanellus vanellus* | - | 67.80 | 34.69 | - | -0.07 | -0.0782 | 12.50 | -0.9915 | 0.5371 | - | - | - | - | - | - |
