# Supplementary material for: Colonial, more widely distributed and less abundant bird species undergo wider population fluctuations independent of their population trend
Source: PLoS One. 2017 Mar 2;12(3):e0173220. doi: 10.1371/journal.pone.0173220 (PMC5333898; doi:10.1371/journal.pone.0173220)
Supplement: S2 Table — (DOCX) [file pone.0173220.s002.docx]

**S2 Table.** Relationships between the magnitude of population fluctuations and abundance, total breeding range, coloniality and population trend of European breeding bird species. The magnitude of population fluctuations was the response variable in a phylogenetic generalized least square regression model.

| Term | Estimate (SE) | *t* | *P* |
| --- | --- | --- | --- |
| Intercept | 1.168 (0.042) | 27.56 | < 0.0001 |
| Abundance | -0.066 (0.007) | -8.99 | < 0.0001 |
| Total range | 0.010 (0.002) | 3.92 | 0.00012 |
| Coloniality | 0.041 (0.015) | 2.77 | 0.0061 |
| Population trend | 0.051 (0.026) | 1.98 | 0.049 |

Phylogenetic relations among species and the number of populations used to estimate population fluctuations in each species were controlled in the analysis (see Statistical analysis for details). The model had the statistics: *F* = 28.75, adj-*r*^2^ = 0.329, *N* = 227, *P* < 0.0001, λ = 0.367.
